# Bats host the most virulent—but not the most dangerous—zoonotic viruses

**DOI:** 10.1073/pnas.2113628119

**Published:** 2022-03-29

**Authors:** Sarah Guth, Nardus Mollentze, Katia Renault, Daniel G. Streicker, Elisa Visher, Mike Boots, Cara E. Brook

**Affiliations:** ^a^Department of Integrative Biology, University of California, Berkeley, Berkeley, CA 94720;; ^b^Medical Research Council–University of Glasgow Centre for Virus Research, Glasgow G61 1QH, United Kingdom;; ^c^Institute of Biodiversity, Animal Health and Comparative Medicine, College of Medical, Veterinary and Life Sciences, University of Glasgow, Glasgow G12 8QQ, United Kingdom;; ^d^Centre for Ecology and Conservation, University of Exeter, Exeter TR10 9FE, United Kingdom;; ^e^Department of Ecology and Evolution, University of Chicago, Chicago, IL 60637

**Keywords:** zoonotic viruses, emerging infectious diseases, virulence, death burden, bat viruses

## Abstract

Identifying virus characteristics associated with the largest public health impacts on human populations is critical to informing “zoonotic risk” assessments and surveillance strategies. Efforts to assess zoonotic risk often use trait-based analyses to identify which viral and reservoir host groups are most likely to source zoonoses but have not fully addressed how and why the impacts of zoonotic viruses vary in terms of disease severity (“virulence”), capacity to spread within human populations (“transmissibility”), or total human mortality (“death burden”). We analyzed trends in human case fatality rates, transmission capacities, and total death burdens across a comprehensive dataset of mammalian and avian zoonotic viruses. Bats harbor the most virulent zoonotic viruses even when compared to birds, which alongside bats have been hypothesized to be special zoonotic reservoirs due to molecular adaptations that support the physiology of flight. Reservoir host groups more closely related to humans—in particular, primates—harbor less virulent but more highly transmissible viruses. Importantly, a disproportionately high human death burden, arguably the most important metric of zoonotic risk, is not associated with any animal reservoir, including bats. Our data demonstrate that mechanisms driving death burdens are diverse and often contradict trait-based predictions. Ultimately, total human mortality is dependent on context-specific epidemiological dynamics, which are shaped by a combination of viral traits and conditions in the animal host population and across and beyond the human–animal interface. Understanding the conditions that predict high zoonotic burden in humans will require longitudinal studies of epidemiological dynamics in wildlife and human populations.

## Significance

The clear need to mitigate zoonotic risk has fueled increased viral discovery in specific reservoir host taxa. We show that a combination of viral and reservoir traits can predict zoonotic virus virulence and transmissibility in humans, supporting the hypothesis that bats harbor exceptionally virulent zoonoses. However, pandemic prevention requires thinking beyond zoonotic capacity, virulence, and transmissibility to consider collective “burden” on human health. For this, viral discovery targeting specific reservoirs may be inefficient as death burden correlates with viral, not reservoir, traits, and depends on context-specific epidemiological dynamics across and beyond the human–animal interface. These findings suggest that longitudinal studies of viral dynamics in reservoir and spillover host populations may offer the most effective strategy for mitigating zoonotic risk.

The vast majority of human pathogens are derived from animal populations (1). In response to increasingly frequent zoonotic spillovers and their substantial public health risks (2), there has been a movement to identify the ecological systems and taxonomic groups of animals and pathogens that are most likely to source the next emerging zoonosis in the human population (3–9). However, most of this work has centered on a binary definition of zoonotic risk—whether particular pathogens are capable of infecting humans—without considering how pathogens vary with respect to their impacts on humans after spillover. The ongoing severe acute respiratory syndrome coronavirus 2 (SARS-CoV-2) pandemic has re-emphasized the reality that not all zoonoses pose risks of equal magnitude—some are exceptionally more “dangerous” than others due to the severity of disease they cause (“virulence”) or their capacity to spread within human populations (“transmissibility”), which combined influence the total number of human deaths (“death burden”) (10). Given the extraordinary diversity of both animal hosts and the viruses they harbor, understanding which animal and virus groups are more likely to source dangerous zoonoses is an important public health aim. Many high-profile zoonotic viruses—including Nipah and Hendra henipaviruses; Ebola filovirus; SARS, Middle East respiratory syndrome (MERS), and SARS-CoV-2 coronaviruses; pandemic avian influenzas; West Nile virus; and Eastern Equine encephalitis virus—have emerged from Chiropteran (bat) or avian reservoirs (11). The high number of zoonotic viruses found in bats and birds has been attributed to their large gregarious populations, mobility, ability to colonize anthropogenic environments, and sheer species diversity (7, 11). Nonetheless, the following question remains: are bat- and/or bird-borne viruses disproportionately dangerous?

A recent analysis (10) found that mammalian reservoir hosts most closely related to humans harbor zoonoses of lower impact in terms of mortality relative to more phylogenetically distant hosts. These results were consistent with phylogenetic trends in virulence that have been reported in cross-species pathogen emergences in other systems (12, 13) and likely reflect mismatches in host biology, physiology, and ecology. Notably, order Chiroptera (bats)—one of the more distantly related host orders—had the highest positive effect size on case fatality rate (CFR) in humans. Nevertheless, this analysis considered only directly transmitted viruses and viruses derived from mammalian hosts, despite the existence of several high-profile vector-borne and avian zoonoses (11). In particular, birds occupy a separate taxonomic class from humans—a phylogenetic distance that might correlate with heightened virulence in humans.

In vitro work has suggested that molecular adaptations that support the physiology of flight, a trait unique to bats among mammals, may allow bats to tolerate rapidly replicating viruses that express heightened virulence upon emergence in less tolerant hosts such as humans (14)—thus offering a possible explanation for bat virus virulence. Bats and birds share a suite of convergent flight adaptations—both taxa are remarkably long-lived for their body size and appear to circumvent metabolic constraints on longevity through cellular pathways evolved to mitigate oxidative stress induced by flight (11). These metabolic adaptations are hypothesized to be linked to the evolution of virulent viruses in bats, but they are only typically discussed with respect to their effect on lifespan in birds (15). A few papers have reviewed birds’ role as “special” zoonotic reservoirs (11, 16), but the virulence of avian zoonoses remains largely unexplored. Nonetheless, although the most virulent zoonotic viruses may garner the most publicity, these pathogens are not necessarily the most dangerous to human health. Rather, human health is most impacted by viruses that cause large volumes of cases and deaths (burden). Although some viruses such as Ebola and rabies are associated with both high CFRs and burden in the human population, pandemic viruses are often characterized by relatively low CFRs but high human transmissibility. The 2009 H1N1 influenza pandemic was estimated to have caused 60.8 million cases and more than 12,000 deaths in the United States alone with a CFR of less than 1% (17), and as of 9 July 2021, SARS-CoV-2 has caused over 185 million cases and 4 million deaths worldwide with a CFR of just 2.2% (18). To prevent the next zoonotic pandemic, it is important to think beyond the individual measures of zoonotic capacity, virulence, and transmissibility to consider collective burden on public health.

We applied generalized additive models (GAMs) to a dataset of mammalian and avian zoonotic viruses to identify reservoir host and viral traits predictive of the 1) CFR, 2) capacity for forward transmission, and 3) death burden induced by infections in the human population—with the goal of characterizing sources of zoonotic viruses that pose the greatest “danger” to global health. Our work builds on a small body of analyses that have begun to explore variation in the virulence and between-human transmissibility of zoonotic viruses (4, 19–21). We provide an analysis of burden and the largest sample size—with trends examined across the majority of known zoonotic viruses. We hypothesized that birds—given their capacity for flight and phylogenetic distance from humans—might rival bats for the association with the most virulent zoonotic viruses. However, we did not expect bats or birds to be responsible for the greatest burden on global health, instead anticipating high burden to be largely a function of viral traits and association with reservoir orders that harbor less virulent, more transmissible viruses.

## Results

Drawing from existing databases (7, 10, 22), we compiled a dataset of all mammalian and avian zoonotic virus species that met a strict definition of zoonotic—requiring a post-1950 record of natural human infection confirmed by PCR or sequencing and animal-to-human directionality in transmission (*Materials and Methods*, *Constructing the Database*). Virus species linked to multiple independent reservoir groups (e.g., canine and bat rabies) or those which spillover to humans both directly from their reservoir and through bridge hosts (e.g., Nipah virus) were subdivided into separate entries for each unique transmission chain ending in spillover, creating a final dataset of 89 viruses with a total of 93 transmission chains (*SI Appendix*, Table S1). We then applied GAMs to assess predictors (*SI Appendix*, Table S7) of the following three metrics of zoonotic risk: global estimates of CFRs in humans (proxy for virulence), capacity for forward transmission within the human population ranked on a four-point scale (human transmissibility), and post-1950 cumulative death counts (death burden) (*SI Appendix*, Fig. S1). We used both Akaike information criterion (AIC)-maximization model selection (results reported in the main manuscript) and automated term selection by double penalty smoothing (*SI Appendix*, Table S8), and we found that all key results were consistent across the two variable selection techniques.

### Predictors of Human CFRs.

**Fig. 1.**
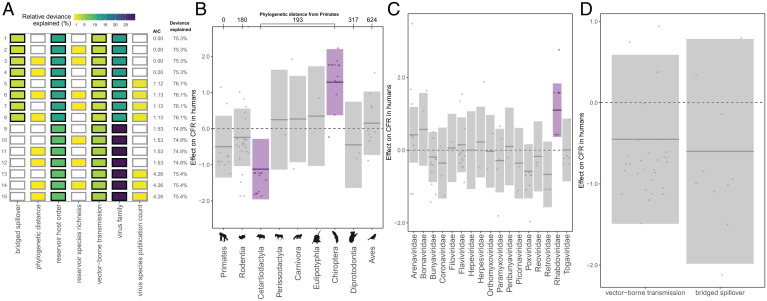
Predictors of global CFR estimates. (*A*) Top 15 models ranked by AIC. Rows represent individual models, and columns represent predictor variables. Cells are shaded according to the proportion of deviance explained by each predictor. Cells representing predictor variables with a *P* value significance level of <0.1 are outlined in black. (*B*–*D*) Effects present in the top model, namely, reservoir host group, virus family, vector-borne transmission, and bridged spillover. Lines represent the predicted effect of the x-axis variable when all other variables are held at their median value (if numeric) or their mode (if categorical). Shaded regions indicate 95% CIs by SE, and points represent partial residuals. An effect is shaded in gray if the 95% CI crosses zero across the entire range of the predictor variable; in contrast, an effect is shaded in purple and considered “significant” if the 95% CI does not cross zero. Full model results are outlined in *SI Appendix*, Table S5A. (*B*) Reservoir host groups are ordered by increasing cophenetic phylogenetic distance from Primates (in millions of years), as indicated on the *Top* axis.

In our virulence analysis, we observed a left-skewed distribution of CFRs, with 33.7% of virus species linked to no fatalities (0% CFR) and more than half (57.8%) linked to a CFR of less than 10% (*SI Appendix*, Fig. S2). Bat reservoirs contributed more than two-thirds (68.8%) of the identified viruses with CFRs higher than 50%. The top selected GAM to predict global estimates of CFR in humans—across the 87 unique zoonotic transmission chains for which at least 2 human cases have been recorded—explained 75.3% of the deviance and included reservoir host group, virus family, bridged spillover, and vector-borne transmission (Fig. 1 and *SI Appendix*, Table S5A). Consistent with previous work (10) and the hypothesis that bats are special zoonotic reservoirs, order Chiroptera had the largest positive effect size on CFR in humans (Fig. 1*B*). The top selected model predicted a CFR of 65.6% for zoonotic viruses derived from order Chiroptera, representing a more than 50% increase from the next highest predicted CFR (*SI Appendix*, Fig. S3). Nevertheless, overlapping confidence intervals for both CFR predictions (*SI Appendix*, Fig. S3) and effect sizes (Fig. 1*B*) indicated that without larger sample sizes, we cannot eliminate all uncertainty regarding the virulence of bat viruses relative to viruses from reservoir groups with very few known zoonotic viruses. Contrary to our flight hypothesis, avian reservoirs were not similarly associated with disproportionately virulent zoonoses; order Aves had a neutral effect size on human CFR that was not significant. Order Cetartiodactyla—which in our dataset, included on domesticated animal species (i.e., cattle, pigs, and camels)—had the largest negative effect size on CFR.

Past analyses have observed that particular viral families associate nonrandomly with particular host groups (10, 23), suggesting that virus taxonomy may underlie trends in virulence across reservoir orders. For example, the high number of virulent bat-borne zoonoses (*SI Appendix*, Fig. S2) may be entirely a result of the virus groups that preferentially infect bats, rather than the bats themselves. However, here, reservoir host group and virus family significantly predicted CFR within the same models (Fig. 1*A*), indicating that both reservoir and virus taxa contributed to the observed variation in virulence. Chiroptera had the highest positive effect size on CFR despite being associated with virus families that ranged from the most (Rhabdoviridae) to least (Coronaviridae) virulent (Fig. 1*C*). Removing the 100% fatal lyssaviruses (*n* = 5) from the dataset resulted in large reductions in the CFR predicted for bat-borne zoonoses (*SI Appendix*, Fig. S5), although order Chiroptera still had the highest and most significant positive effect size on CFR—indicating that observed patterns were not driven by rabies alone (*SI Appendix*, Fig. S4 and Table S6A).

Previous work has demonstrated a positive correlation between reservoir host phylogenetic distance from humans and the CFRs of zoonoses derived from those reservoirs (10); in our analysis, however, reservoir host group phylogenetic distance from Primates was not correlated with CFR, dropping entirely from the top ranked model and not ranking significantly in any of the top 15 selected models (Fig. 1*A*). The combined effect of reservoir host group and virus family as predictor variables in the same model likely overwhelmed any correlation between host phylogeny and CFR, particularly given the lack of granularity in our phylogenetic distance variable, based on a time-scaled phylogeny, which produced only six unique distance values across nine host groups, with Chiroptera and four of the other mammalian orders clustering at a single distance level (*Materials and Methods*). Nevertheless, trends in effect size on CFR (Fig. 1*B*) and predicted CFR (*SI Appendix*, Fig. S3) across reservoir host groups suggest that, in general, virulence increases with phylogenetic distance, but this positive correlation may collapse at “extreme” distances.

To test whether these results held across a larger sample size, we ran a CFR analysis that included viruses that met a more lenient definition of zoonotic—specifically, viruses with only serological evidence of infection in humans, viruses that have only caused human infections in laboratory settings, and viruses for which only one human case has been recorded—increasing our dataset to 121 virus species with a total of 126 unique zoonotic transmission chains (*SI Appendix*, Fig. S6 and Table S6B). This supplementary analysis echoed the results from our first analysis of global CFR estimates (*SI Appendix*, Fig. S6*A*)—both reservoir and virus taxonomy contributed to the observed variation in CFR and Chiroptera had the highest positive effect size on CFR, whereas Aves had a neutral nonsignificant effect (*SI Appendix*, Fig. S6*B*).

To assess whether CFR trends might be biased by viruses’ geographic ranges (e.g., differences in health care infrastructure and case ascertainment), we tested whether gross domestic product per capita (GDP per-capita) significantly predicted country-specific CFR estimates—calculated from death and case counts in countries that have reported the largest outbreaks of each given virus species, with up to 3 country estimates for each species for a total of 119 estimates across the 87 unique zoonotic transmission chains. First, we modeled all 119 country-specific CFR estimates separately to test whether GDP per-capita predicts country-level variation in CFR (*SI Appendix*, Fig. S7 and Table S6C). We then modeled GDP per-capita and country CFR estimates aggregated at the level of the 87 unique zoonotic transmission chains (*SI Appendix*, Fig. S8 and Table S6D). In both analyses, GDP per-capita was not significant in any of the top models, often dropping entirely during model selection (*SI Appendix*, Figs. S7*A* and S8*A*), suggesting that viruses’ geographic ranges most likely do not bias Fig. 1 trends. Nevertheless, as with the supplementary analyses presented in *SI Appendix*, Figs. S4 and S6, both analyses of the country CFR estimates echoed all key results presented in Fig. 1.

### Predictors of Transmissibility within Human Populations.

**Fig. 2.**
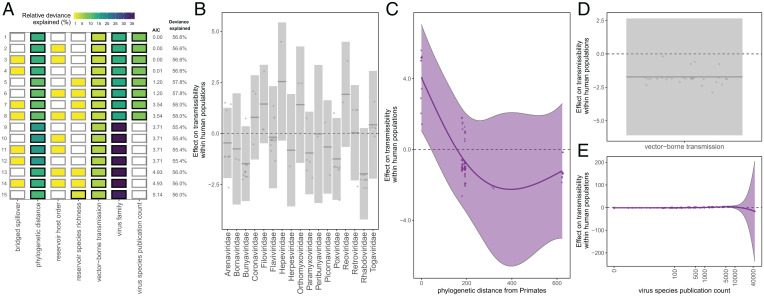
Predictors of capacity for forward transmission within the human population following zoonotic spillover. (*A*) Top 15 models ranked by AIC. Rows represent individual models and columns represent predictor variables. Cells are shaded according to the proportion of deviance explained by each predictor. Cells representing predictor variables with a *P* value significance level of <0.1 are outlined in black and otherwise outlined in gray. (*B*–*E*) Effects present in the top model, namely, virus family, reservoir group phylogenetic distance from Primates, vector-borne transmission, and log-transformed virus species publication count. Lines represent the predicted effect of the x-axis variable when all other variables are held at their median value (if numeric) or their mode (if categorical). Shaded regions indicate 95% CIs by SE, and points represent partial residuals. An effect is shaded in gray if the 95% CI crosses zero across the entire range of the predictor variable; in contrast, an effect is shaded in purple and considered significant if the 95% CI does not cross zero. Full model results are outlined in *SI Appendix*, Table S5B.

We found that most zoonotic viruses (71.3%) have not been reported to transmit within the human population following spillover (i.e., transmissibility rank = 1, or R_0_ = 0) (*SI Appendix*, Fig. S9). Only 14.9% of virus species had demonstrated capacity for endemic transmission among humans, of which the majority (61.5%) were sourced from Primates. The top selected GAM to predict the ordinal rank of transmissibility within human populations—across the 87 unique zoonotic transmission chains for which at least 2 human cases have been recorded—explained 56.6% of the deviance and included virus family, the phylogenetic distance between each viruses’ reservoir host group and Primates, vector-borne transmission, and the virus species publication count (Fig. 2 and *SI Appendix*, Table S5B). Transmissibility declined with phylogenetic distance from Primates, but the estimated trend was highly uncertain (Fig. 2*C*). We therefore reran the analysis with reservoir host group as the only host taxonomic predictor (excluding the phylogenetic distance variable). This model explained 55.9% of the deviance and identified Primates as the only host order significantly associated with heightened transmissibility in humans, suggesting that this group is the primary driver of the phylogenetic trend observed in the top selected model (*SI Appendix*, Fig. S10*A* and Table S6E).

### Predictors of Post-1950 Death Burden in the Human Population.

**Fig. 3.**
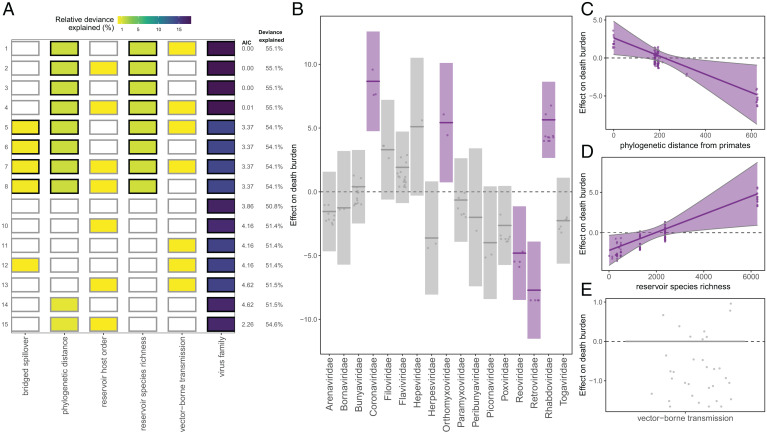
Predictors of post-1950 death burden, excluding the virus species publication count predictor. See *SI Appendix*, Fig. S12 for inclusion. (*A*) Top 15 models ranked by AIC. Rows represent individual models and columns represent predictor variables. Cells are shaded according to the proportion of deviance explained by each predictor. Cells representing predictor variables with a *P* value significance level of <0.1 are outlined in black and otherwise outlined in gray. (*B*–*E*) Effects present in the top model, namely, virus family, reservoir group phylogenetic distance from Primates, reservoir group species richness, and vector-borne transmission. Lines represent the predicted effect of the x-axis variable when all other variables are held at their median value (if numeric) or their mode (if categorical). Shaded regions indicate 95% CIs by SE, and points represent partial residuals. An effect is shaded in gray if the 95% CI crosses zero across the entire range of the predictor variable; in contrast, an effect is shaded in purple and considered significant if the 95% CI does not cross zero. Full model results are outlined in *SI Appendix*, Table S5C.

For our death burden analysis, we modeled the total number of deaths resulting from a given zoonosis recorded worldwide since 1950 (and up until 7 March 2021). In cases where our death count could only begin after 1950, either because a zoonosis first emerged in humans after 1950 or because reliable death records were only available for a subset of the timeline, we standardized analyses by including an offset for the number of years over which the death counts were recorded. The raw death count distribution was highly left-skewed, with 39.8% of virus species linked to zero deaths and more than half (62.5%) linked to fewer than 50 deaths (*SI Appendix*, Fig. S12). We observed significant overdispersion in death counts, even when standardized by the number of years over which the deaths were recorded, with deaths per year ranging from 0 to almost 2 million for SARS-CoV-2. Just 2 viral predictors—virus family and species publication count—explained most of the variation in death burden among the 93 zoonotic transmission chains across all the top GAMs (*SI Appendix*, Fig. S13*A*). Host predictors explained a very low percentage of the variation in death burden across all the top selected models, often dropping entirely during term selection. Virus species publication count tempered virus family effects (*SI Appendix*, Fig. S13*C*) because virus species with high death burdens were also associated with high publication counts, likely because high death burdens motivate increased research efforts. In contrast, there was little evidence that only poorly studied viruses were limited to unusually low death burdens, implying that a lack of diagnostic effort is not a major driver of low death burdens in our data (*SI Appendix*, Fig. S13*C*). After excluding the virus species publication predictor, we found that Coronaviridae, Orthomyxoviridae, and Rhabdoviridae had the highest positive effect sizes on death burden, driven by the SARS-CoV-2, the influenza A transmission chains, and Rabies virus, respectively (Fig. 3*B* and *SI Appendix*, Table S5*C*). With virus publication count removed, the top four models included two reservoir traits—phylogenetic distance from Primates and species richness—as significant predictors. Reservoir groups most closely related to Primates were associated with heightened death burdens relative to more distantly related reservoirs, consistent with results from our transmissibility analyses that indicated that reservoirs most closely related to Primates harbored more transmissible viruses (Fig. 3*C*). Reservoir species richness positively correlated with death burden, as we would expect given that species richness has been found to correlate with the number of viruses associated with a given reservoir order (Fig. 3*D*) (7). However, both reservoir predictors explained a small fraction of the variation in death burden relative to virus family, confirming that death burden is largely a function of viral traits (Fig. 3*A*).

**Fig. 4.**
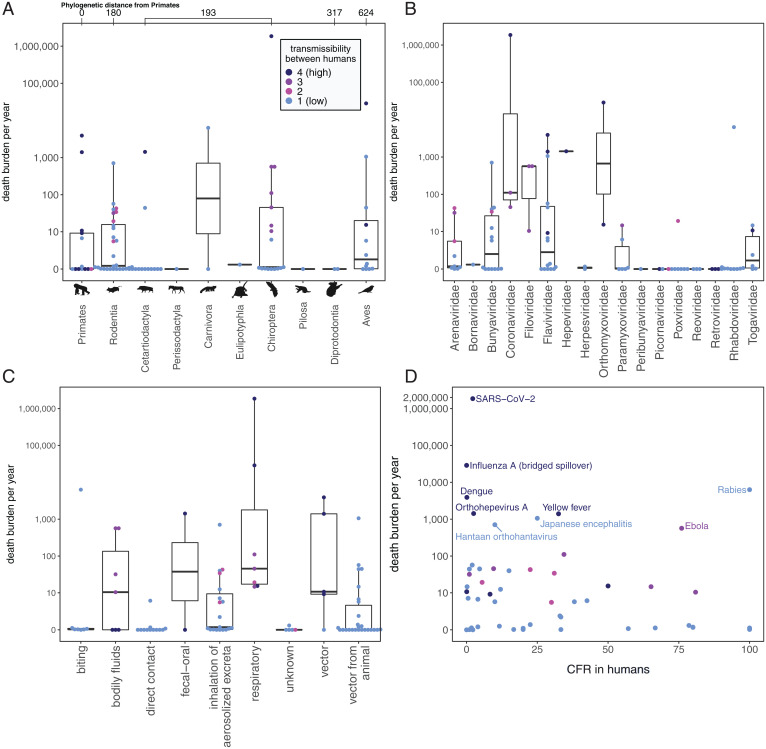
Death burden per year (cumulative post-1950 death counts divided by the length of reporting time), grouped by reservoir host group (*A*), virus family (*B*), primary transmission route (*C*), and CFR in humans (*D*). Colors indicate transmissibility between humans, with “1” indicating the lowest level of transmission (i.e., no recorded forward transmission in human population postspillover) and “4” indicating the highest level of transmission (i.e., record of endemic transmission in human populations postspillover). (*A*) Reservoir host groups are ordered by increasing cophenetic phylogenetic distance from Primates (in millions of years), as indicated on the *Top* axis.

Although some reservoir groups—bats, primates, rodents, and birds—have sourced more high-burden viruses than others (Fig. 4*A*), both our model results and raw data suggested that high burden viruses appeared to be a function of viral traits, not the reservoirs themselves. No single reservoir stood out as a consistent source of high-burden viruses, with every reservoir that harbors high-burden viruses also harboring substantially more viruses that cluster at the lowest death burdens (Fig. 4*A*). This was not the case for virus family (Fig. 4*B*) or primary transmission route (Fig. 4*C*); Coronaviridae and Orthomyxoviridae and a respiratory transmission route were associated only with high-burden zoonotic viruses. In general, the viruses linked to the lowest death burdens were associated with the lowest transmission capacity. As a deviation from this trend, Primates—which our models indicate harbor the most transmissible, but generally less virulent, zoonotic viruses—harbored several highly transmissible viruses with low death burdens (Fig. 4*A*).

The highest death burdens were overall associated with zoonotic viruses that are less virulent but highly transmissible in human populations (Fig. 4*D*). Respiratory pathogens with a capacity for human-to-human transmission have often incurred massive burdens over short timeframes as a result of rare, but catastrophic spillover events that spark widespread transmission in humans. Critically, although our dataset included only 6 viruses with respiratory droplets as a primary transmission route—SARS-CoV-1, SARS-CoV-2, MERS-CoV, influenza A, Nipah, and monkeypox—these viruses accounted for more than 85.9% of the deaths recorded for the 86 viruses in our death burden analysis, highlighting respiratory transmission as a high-risk zoonotic trait. However, these data were derived from a notably small sample size, as three of the six respiratory viruses have caused only a single major epidemic. There was also substantial variation among these respiratory viruses, with the death burdens associated with SARS-CoV-1 and SARS-CoV-2 differing by more than 2.5 million people.

## Discussion

A key insight from our work is that bats harbor the most virulent zoonotic viruses relative to other mammalian and avian reservoirs (*SI Appendix*, Fig. S2). Given that birds represent the only other flying vertebrates and that flight adaptations are hypothesized to influence the evolution of viruses virulent to humans in bat reservoirs (11), we expected avian viruses to similarly be associated with heightened CFRs in humans. However, we found that only order Chiroptera had an exceptionally high positive effect size on CFR in humans, whereas Aves had a neutral nonsignificant effect. It is of course possible that we observed this association between Chiroptera and high CFRs in part because low-virulence zoonotic viruses have gone undetected in bat reservoirs; however, other poorly studied reservoirs are not comparably associated with heightened virulence, suggesting that detection bias cannot explain our results. Like CFR, transmissibility in humans was also correlated with reservoir traits, but in this case, Primates—the reservoir group most closely related to humans—sourced the zoonotic viruses with the highest capacities for forward transmission in human populations. Although a combination of both virus and reservoir taxonomy predicted virulence and transmissibility, death burden did not correlate with any reservoir group and instead was a function of viral traits. Nevertheless, our data indicated that mechanisms driving high death burdens are diverse and often contradict trait-based predictions. Several high-profile zoonotic viruses linked to significantly higher death burdens than we would expect based on their capacity for forward transmission in the human population (Fig. 4*D*), suggesting that death burden is highly dependent on both the contact rate at the human–animal interface and epidemiological dynamics within the human population—factors that are not fully captured by the broad explanatory variables considered in trait-based analyses.

Evolution of virulence theory typically assumes a tradeoff between virulence (death rate due to infection) and transmission rate on the basis that although high within-host growth rates increase infectiousness, they also increase damage to the host, increasing virulence and thus shortening the infectious period and reducing opportunities for future transmission (24, 25). Critically, CFR is not equivalent to virulence, but instead, it is a proxy that can be reliably quantified. As defined by Day (26), CFR is a function of both pathogen virulence (α) and clearance rate (σ), in which CFR=α/(α+σ). Thus, virulent pathogens (high α) with high clearance rates (high σ)—e.g., acute, short-lived infections such as Chikungunya virus (27)—could produce low CFRs. In contrast, less virulent pathogens (low α) with low clearance rates (low σ)—e.g., persistent infections such as HIV (28)—could produce high CFRs. Nevertheless, in our data, we observed a relationship between CFR and transmissibility in humans that roughly supports the fundamental theoretical tradeoff between virulence and transmission rate (*SI Appendix*, Fig. S11). Viruses causing the highest CFRs in humans (>75% CFR) clustered in the lower right corner with the lowest capacity for forward transmission in the human population, implying maladaptive virulence. Conversely, the least virulent viruses (0% CFR) clustered at either the lowest transmission capacity—likely indicative of poor compatibility with humans—or the highest transmission capacity—suggesting transmission uninhibited by virulence.

The surprisingly low virulence of avian zoonotic viruses in contrast to bat-borne viruses may reflect the extreme phylogenetic distance that separates birds from Primates. In our previous analysis, we found that mammalian reservoir hosts most closely related to humans harbor less virulent zoonotic viruses relative to more distantly related mammalian hosts such as bats (10). This positive correlation between reservoir phylogenetic distance from humans and viral virulence is consistent with trends that have been reported in cross-species pathogen emergences in other systems (10, 12, 13) and likely reflects maladaptive virulence resulting from mismatches in host biology, physiology, and ecology. Clearly, although bats are distantly related to humans, they are still mammals, whereas birds occupy a separate taxonomic class. It is possible that the positive correlation between phylogenetic distance and virulence collapses at distances beyond mammals because viruses are expected to have a limited capacity to replicate in host environments that are very different from that of their reservoir, leading to nonhost resistance (29, 30). Phylogenetic distance dropped from all CFR models likely due to a lack of granularity in our phylogenetic distance data, which described reservoir host cophenetic distance from Primates on a time-scaled phylogeny (7), producing only six unique distance values across all of the reservoir groups in our database. Trends across reservoir host groups overall support the hypothesis that the positive correlation between phylogenetic distance and virulence collapses at extreme distances. Nevertheless, more studies are needed to parse the effect of phylogenetic distance on virulence trends in animal-to-human spillovers. The time-scaled phylogeny represents the only available phylogeny that includes both mammals and birds. Future studies would benefit from developing additional phylogenies of mammalian and avian reservoirs, which prioritize immunological or physiological traits that may more accurately proxy virologically relevant differences in host environments.

Chiroptera represented an outlier among distantly related reservoirs, with an undeniably positive effect size on CFR more than triple that recovered for any other mammalian order. Consistent with the hypothesis that bats represent a special viral reservoir (31), the order Chiroptera does appear to harbor zoonotic viruses that are uniquely virulent upon spillover to humans, even when considering virulence effects that might be attributed to their phylogenetic distance from Primates. In bats, flight adaptations have been linked to viral tolerance, which previous work suggests may select for high growth rate viruses that could manifest as virulent upon emergence in less tolerant hosts such as humans (14). Notably, bats experience limited morbidity or mortality from intracellular infections with only a few known exceptions (31–34). Conversely, although birds harbor several zoonotic viruses that are virulent in humans such as highly pathogenic avian influenza (HPAI), West Nile, and equine encephalitis viruses, only some avian species are tolerant of these infections—many avian species experience morbidity and mortality (35). Bats and birds are expected to experience similar selective pressures from flight; they have been found to incur comparable energetic costs while flying, despite different forms and physiologies (15, 36). However, the two taxonomic groups, within disparate vertebrate classes, may have responded differently to these selective pressures. Specifically, there is a possibility that bats evolved cellular pathways that protect against both aging and immunopathology, whereas birds evolved pathways that only protect against aging. For example, bats have been found to host a suite of cellular-level anti-inflammatory adaptations—including enhanced cellular autophagy and downregulated signaling pathways linked to the induction of inflammatory antiviral defenses—which may both mitigate cellular damage induced by bat metabolism and inhibit immunopathology incurred upon viral infection (31, 37–41). On the other hand, birds may rely primarily on systemic antioxidant responses (42), which mitigate oxidative stress but do not interact so tightly with cellular-level processes that impact viral pathology. Critically, birds appear to be missing anti-inflammatory protein tristetraprolin (43), and immunopathology is often the cause of death in birds that die from viral infections such as HPAI and West Nile virus (35). Differences between mammalian and avian immune systems may additionally play a role in their differing infection outcomes. The immune system is broadly conserved in amniotes, but some avian immunological features diverge from those of bats and other mammals. Notably, birds lack lymph nodes and instead develop B cells in a specialized lymphoid organ, the bursa of Fabricius; have heterophil in their white blood cells as opposed to neutrophil; and produce only three classes of immunoglobulin in contrast to the five produced by mammals (11). Nevertheless, the differing effects of Chiropteran and avian metabolic adaptations on viral tolerance and viral evolution remain largely uncharacterized, and more basic research in this field is needed (44).

Order Cetartiodactyla had the largest negative effect size on CFR, but notably, Cetartiodactyl hosts in our dataset included only domesticated animal species—cattle, pigs, and camels. The long coexistence of domestic animals and humans likely facilitated the increased research effort for this clade, which have may have led to the greater detection of low virulence zoonoses in domestic animal species. A long history of domestic animal-human coexistence may also have supported the development of preexisting human immunity to some livestock diseases, resulting in lower virulence infections.

We found that both reservoir host and virus taxonomy predict the virulence and transmissibility of a virus in the secondary human host, consistent with the expectation that a virus evolves virulence to maximize reproduction in its reservoir population (45). The optimal balance between virulence and transmission depends on how the reservoir host population responds to the virus (the host selective pressure), which is determined by the ecological, physiological, and biological traits of the reservoir. Although we identified special reservoirs of virulent and transmissible zoonotic viruses, we found that the human death burden incurred by viral zoonoses does not correlate with any one reservoir host order, including bats, and instead is a function of viral traits. Our data demonstrate that mechanisms driving high death burdens are diverse and often contradict trait-based predictions. High death burdens have resulted from rare spillover events of highly transmissible viruses that spread widely in the human population; small, but frequent spillovers of the least transmissible viruses; and historically low-burden pathogens that take off given the right ecological and evolutionary conditions. This suggests that, ultimately, death burden depends on epidemiological circumstances, which should be shaped, not by reservoir host traits, but by a combination of viral traits and conditions in the animal host population and across and beyond the human–animal interface. Notably, the pandemic spread of SARS-CoV-2 can be attributed to its highly effective respiratory transmission between humans, a trait linked to its identity within Coronaviridae, rather than its bat origins (indeed, CoVs demonstrate gastrointestinal tropism in bat reservoirs) (46).

However, several outliers demonstrated that a capacity for forward transmission in human populations does not always predict death burden; it is critical to also consider epidemiological dynamics across and beyond the human–animal interface. Less-transmissible viruses can accumulate large death burdens over many small, but frequent spillovers, particularly in systems in which humans regularly interact with animal reservoirs. Rabies, Hantaan (HTNV), and Japanese encephalitis viruses have been associated with some of the highest death burdens induced by viral zoonosis despite lacking forward transmission in human populations (Fig. 4*D*). This is likely because these viruses spill over to humans from animal host populations that live among human communities—rabies burden is largely driven by spillover from endemic circulation in domestic dogs (47), HTNV spills over from striped field mouse (*Apodemus agrarius*) populations that inhabit agricultural fields (48), and Japanese encephalitis is amplified via domesticated pigs (49). Outbreaks in these spillover host populations source human infections that are dead ends for further transmission but add up to large numbers. Emphasizing the importance of understanding system-specific dynamics, HTNV had a death burden more than 18 times greater than the combined death burden of all 10 other rodent-borne hantaviruses in our dataset, most likely because other rodent reservoirs of hantaviruses tend to overlap less with human populations (48). Furthermore, zoonotic viruses that have historically been low burden pathogens can unexpectedly cause high death burdens in the case of virus evolution or unique epidemiological circumstances (50). For example, Ebola virus first emerged in humans in 1976, causing deadly, but local outbreaks up until late 2013, when suddenly, emergence in a region with dense and interconnected human populations, coupled with virus adaptation (51), allowed an Ebola virus spillover event to spark a transnational epidemic that in just 2 y caused more than 6.5 times the total number of deaths recorded from 1976 to 2013 (50, 52). These outliers suggest that understanding epidemiological dynamics—within wildlife populations and across and beyond the human–animal interface—in specific systems is a critical component of predicting death burden and, consequently, danger to human health.

Over the course of the last decade, a significant amount of funding and research effort has been dedicated to identifying correlates of zoonotic risk, often with a long-term aspiration of identifying ways to anticipate and prevent emerging zoonoses in the future (53–55). This research increasingly prioritizes viral discovery over longitudinal studies of epidemiological dynamics and targets animal populations such as bats that have been identified as key zoonotic reservoirs. Although our analysis corroborates the hypothesis that bats are a special reservoir for virulent zoonotic viruses, we also demonstrate that viral traits—not bat reservoirs—pose the greatest danger to human health. We argue that burden, which does not correlate with any animal reservoir and instead appears to be a function of transmission conditions to and within the human population, more correctly approximates danger to human health than does virus virulence. Although reservoir and viral traits can predict zoonotic capacity, virulence, and transmissibility, death burden is dependent on system-specific epidemiological dynamics, which are shaped by a combination of viral traits and conditions in the animal host population and across and beyond the human–animal interface. Thus, understanding and controlling the mechanisms that drive high death burdens in humans—high rates of human–animal contact and/or epidemiological dynamics in the human population that allow discrete spillover events to trigger human epidemics—requires longitudinal surveillance of specific zoonotic or potentially zoonotic viruses in both animal and human populations. There is a pressing need for more longitudinal studies of transmission dynamics in human and wildlife populations to better understand and prevent the epidemiological conditions that cultivate the most dangerous cases of zoonotic viral emergence.

## Materials and Methods

### Constructing the Database (*SI Appendix*, Fig. S1).

We curated a comprehensive dataset of mammalian and avian zoonotic viruses—and the taxonomic orders of the reservoir hosts from which they were derived—from published databases (7, 10, 22). Reservoirs were defined as the primary host species that is responsible for maintaining zoonotic transmission. Using the information provided in these databases and supplementing with literature searches, we extracted viruses that met a strict definition of zoonotic, requiring at least one published human infection in which the virus species was confirmed by PCR, sequencing, or isolation as well as evidence of animal-to-human directionality in transmission (*SI Appendix*, *Exclusion criteria*). With this strict inclusion criteria, we compiled 89 unique virus species (*SI Appendix*, Table S1).

For each virus–reservoir association, we collected both human CFR as a proxy for virulence and the cumulative global death count as a proxy for burden on the human population. For CFR, we collected two estimates. First, we recorded existing estimates of global CFRs from the literature, calculating averages when ranges were reported. Second, for each virus species, we calculated country-specific CFRs from death and case counts in countries that have reported the largest outbreaks of that virus—to assess and account for potentially confounding effects of regional differences in health care and overall infrastructure (*SI Appendix*, *SI analyses*). For our death burden response variable, we collected the total number of deaths recorded across the world since 1950. In many cases, our death count began after 1950, either because a zoonosis first emerged in humans after 1950 or reliable death records were only available for a subset of the timeline. To standardize, we added a variable for the number of years over which death counts were recorded to use as an offset in our models. Death and case counts were derived, when available, from the Global Infectious Diseases and Epidemiology Network (56)—which contains outbreak data from case reports, government agencies, and published literature records—and supplemented with literature searches. We additionally ranked each zoonosis’ capacity for transmission within human populations—a correlate of R_0_—on a four-point scale (10). All variable descriptions are provided in *SI Appendix*, Table S4.

Drawing from previously published databases (7, 10), we collected seven variables (*SI Appendix*, Table S7) that we hypothesized might predict observed variation in human CFR, capacity for transmission within human populations, and death burden. Given published correlations between phylogenetic distance and virulence in cross-species spillovers (10, 12, 13, 57, 58), we included the reservoir host group cophenetic distance from Primates. We considered both reservoir host and virus taxonomy, recording host order and virus family. However, only 10 avian zoonoses were distributed across several avian reservoir host orders. To test our hypotheses regarding avian zoonoses, we addressed this small sample size by aggregating avian reservoir orders into a single Aves group, while maintaining separate host orders for the mammalian reservoirs. Given that the number of zoonoses harbored by a reservoir group appears to correlate with species diversity within that group (7), we hypothesized that species diversity might influence reservoir effect size on CFR in humans; thus, we included reservoir species richness, which we derived from the Catalogue of Life using version 0.9.6 of the taxize library in R (7, 59), taking the sum of values across bird orders for the Aves reservoir group. We defined a “spillover type” variable to account for the zoonotic transmission chain of each virus, distinguishing between zoonoses that jump into humans directly from the reservoir population and those that spillover to humans from bridge hosts (10). Although the majority of zoonoses were linked to single zoonotic transmission chains, there were a few exceptions with both direct and bridged spillover. For example, zoonotic influenza A virus and Nipah virus (60, 61) have spilled over into the human population directly from their avian and bat reservoirs, respectively, as well as from domestic pig bridge host populations. In such cases, each spillover type (i.e., transmission chain) was entered separately in the database. We included an additional binary variable that identified whether viruses were vector borne, as both theory (24) and previous analyses (19, 20) have suggested a relationship between vector-borne transmission and virulence. Finally, as has been done in other similar analyses, we included virus species publication count to account for any potential publication bias (3, 10, 58).

To pair with our country-specific CFR data, we collected an eighth predictor variable—GDP per-capita—as a proxy for geographical differences in the quality of health care and epidemiological control measures.

We additionally collected, for each virus species, the transmission route that contributes the majority of human infections, extending data published by Brierley et al. (19). We then assessed trends in death burden across transmission types, hypothesizing that density-dependent transmission, as a characteristic of transmission via respiratory droplets, would be associated with the highest death burdens in human populations.

### Statistical Analysis.

Given the nonnormal distribution of our data, expected nonlinear relationships, and nested data structures within our predictor variables (62), we applied GAMs in the mgcv package in R (63) to assess predictors of CFR, transmissibility, and death burden in human populations. Rather than manually specifying higher order polynomial functions, GAMs permit the use of smooth functions to capture nonlinear relationships between response and predictor variables (62, 63). We fit continuous variables (i.e., reservoir group species richness and phylogenetic distance from Primates, and virus species publication count) as smoothed effects, and all binary (i.e., vector-borne status and spillover type) and categorical (i.e., reservoir order and virus family) variables as random effects, as has been done in previous analyses (3, 4, 7, 10). For variable selection, we ran all possible model combinations, ranked by AIC, and selected the models with the lowest AIC values. We confirmed our results by rerunning variable selection with automated term selection by double penalty smoothing. This method bypasses AIC-maximization procedures by constructing an additional penalty for each GAM smooth function, effectively removing terms without predictive power, and has been recognized as superior or comparable to alternative approaches (64). We set an effective degree of freedom cutoff of 0.001 to identify which terms had been penalized and effectively removed from the model (3). The validity of all models was checked using standard methods implemented in the mgcv library (63).

We first asked the following: which reservoir host and virus types are associated with elevated CFRs in human populations following spillover? We constructed GAMs in the beta regression family to query the predictive capacity of our predictor variables (*SI Appendix*, Table S7) on CFR in humans. We compressed our CFR range to the beta distribution interval (0,1) by applying the recommended data transformation y¨ = [y′(N−1) + 1/2]N, where N is the sample size (65, 66). For all CFR analyses, we modeled unique zoonotic transmission chains—which we defined as unique reservoir orders and spillover type combinations per virus. As a result, zoonoses with a single reservoir host order and spillover type were modeled as a single CFR entry, whereas those with multiple reservoir orders and/or spillover types (e.g., influenza A and Nipah viruses) were modeled as multiple CFR entries. We excluded five viruses for which only one human case has been recorded (*SI Appendix*, Table S1), deciding that we could not accurately represent a single observation as a CFR. Our final GAM analysis of global CFR estimates included 82 unique virus species with a total of 86 unique zoonotic transmission chains (*SI Appendix*, Table S5A).

We next asked the following: which reservoir host and virus types are associated with an elevated capacity for transmission within human populations? We constructed a GAM in the ocat (ordered categorical data) family to query the predictive capacity of our predictor variables on transmissibility, defining the vector of categorical cut points, θ, to match our four-point ranking scale (*θ =* 1,2,3,4). We again excluded the five viruses for which only one human case has been recorded (*SI Appendix*, Table S1), deciding that we could not accurately determine between-human transmissibility based on a single observation. Thus, like our CFR analysis, our transmissibility analysis included 82 unique virus species with a total of 86 unique zoonotic transmission chains (*SI Appendix*, Table S5B).

Lastly, we asked the following: which reservoir host and virus types are associated with high death burdens in human populations? The death count data demonstrated strong overdispersion (*SI Appendix*, Fig. S11). Thus, we constructed a negative binomial GAM with the scaled observation period (i.e., number of years over which the death count was recorded) as an offset. We considered simpler Poisson GAMs, as well as zero-inflated models, but enhanced residual quantile-quantile plots (67) suggested that these distributions fit poorly. Unlike our CFR analysis, we did not exclude viruses for which only one human case has been recorded. However, we did exclude a single virus species—Rotavirus A—for which we were unable to distinguish between deaths caused by zoonotic strains versus deaths caused by endemic human strains. Thus, our death burden models included 86 zoonotic viruses with a total of 90 transmission chains (*SI Appendix*, Tables S5C and S6F).

## Supplementary Material

Supplementary File

## Data Availability

All data, data references, code, and materials used in the analysis are publicly available in the main text, the supplementary materials, or the following GitHub repository: https://github.com/sguth1993/zoonotic_risk_meta_analysis.

## References

[1] M. E. J. Woolhouse, S. Gowtage-Sequeria, Host range and emerging and reemerging pathogens. Emerg. Infect. Dis. 11, 1842–1847 (2005). 16485468 10.3201/eid1112.050997 PMC3367654

[2] M. Woolhouse, F. Scott, Z. Hudson, R. Howey, M. Chase-Topping, Human viruses: Discovery and emergence. Philos. Trans. R. Soc. Lond. B Biol. Sci. 367, 2864–2871 (2012). 22966141 10.1098/rstb.2011.0354 PMC3427559

[3] K. J. Olival , Host and viral traits predict zoonotic spillover from mammals. Nature 546, 646–650 (2017). 28636590 10.1038/nature22975 PMC5570460

[4] C. Kreuder Johnson , Spillover and pandemic properties of zoonotic viruses with high host plasticity. Sci. Rep. 5, 14830 (2015). 26445169 10.1038/srep14830 PMC4595845

[5] B. A. Han, A. M. Kramer, J. M. Drake, Global patterns of zoonotic disease in mammals. Trends Parasitol. 32, 565–577 (2016). 27316904 10.1016/j.pt.2016.04.007 PMC4921293

[6] A. D. Washburne , Taxonomic patterns in the zoonotic potential of mammalian viruses. PeerJ 6, e5979 (2018). 30519509 10.7717/peerj.5979 PMC6272030

[7] N. Mollentze, D. G. Streicker, Viral zoonotic risk is homogenous among taxonomic orders of mammalian and avian reservoir hosts. Proc. Natl. Acad. Sci. U.S.A. 117, 9423–9430 (2020). 32284401 10.1073/pnas.1919176117 PMC7196766

[8] C. K. Johnson , Global shifts in mammalian population trends reveal key predictors of virus spillover risk. Proc. Biol. Sci. 287, 20192736 (2020). 32259475 10.1098/rspb.2019.2736 PMC7209068

[9] G. F. Albery, D. J. Becker, Fast-lived hosts and zoonotic risk. Trends Parasitol. 37, 117–129 (2021). 33214097 10.1016/j.pt.2020.10.012

[10] S. Guth, E. Visher, M. Boots, C. E. Brook, Host phylogenetic distance drives trends in virus virulence and transmissibility across the animal-human interface. Philos. Trans. R. Soc. Lond. B Biol. Sci. 374, 20190296 (2019). 31401961 10.1098/rstb.2019.0296 PMC6711300

[11] G. Nabi , Bats and birds as viral reservoirs: A physiological and ecological perspective. Sci. Total Environ. 754, 142372 (2021). 33254850 10.1016/j.scitotenv.2020.142372 PMC7505891

[12] B. Longdon, J. D. Hadfield, C. L. Webster, D. J. Obbard, F. M. Jiggins, Host phylogeny determines viral persistence and replication in novel hosts. PLoS Pathog. 7, e1002260 (2011). 21966271 10.1371/journal.ppat.1002260 PMC3178573

[13] M. J. Farrell, T. J. Davies, Disease mortality in domesticated animals is predicted by host evolutionary relationships. Proc. Natl. Acad. Sci. U.S.A. 116, 7911–7915 (2019). 30926660 10.1073/pnas.1817323116 PMC6475420

[14] C. E. Brook , Accelerated viral dynamics in bat cell lines, with implications for zoonotic emergence. eLife 9, e48401 (2020). 32011232 10.7554/eLife.48401 PMC7064339

[15] J. Munshi-South, G. S. Wilkinson, Bats and birds: Exceptional longevity despite high metabolic rates. Ageing Res. Rev. 9, 12–19 (2010). 19643206 10.1016/j.arr.2009.07.006

[16] J. F.-W. Chan, K. K.-W. To, H. Tse, D.-Y. Jin, K.-Y. Yuen, Interspecies transmission and emergence of novel viruses: Lessons from bats and birds. Trends Microbiol. 21, 544–555 (2013). 23770275 10.1016/j.tim.2013.05.005 PMC7126491

[17] CDC, 2009 H1N1 pandemic. *Centers for Disease Control and Prevention* (2019). https://www.cdc.gov/flu/pandemic-resources/2009-h1n1-pandemic.html. Accessed 29 April 2021.

[18] WHO, WHO Coronavirus Disease (COVID-19) Dashboard (2021). https://covid19.who.int/. Accessed 9 July 2021.

[19] L. Brierley, A. B. Pedersen, M. E. J. Woolhouse, Tissue tropism and transmission ecology predict virulence of human RNA viruses. PLoS Biol. 17, e3000206 (2019). 31770368 10.1371/journal.pbio.3000206 PMC6879112

[20] J. L. Geoghegan, A. M. Senior, F. Di Giallonardo, E. C. Holmes, Virological factors that increase the transmissibility of emerging human viruses. Proc. Natl. Acad. Sci. U.S.A. 113, 4170–4175 (2016). 27001840 10.1073/pnas.1521582113 PMC4839412

[21] J. W. Walker, B. A. Han, I. M. Ott, J. M. Drake, Transmissibility of emerging viral zoonoses. PLoS One 13, e0206926 (2018). 30403733 10.1371/journal.pone.0206926 PMC6221319

[22] R. Gibb , Data proliferation, reconciliation, and synthesis in viral ecology. Bioscience 71, 1148–1156 (2021).

[23] S. A. Babayan, R. J. Orton, D. G. Streicker, Predicting reservoir hosts and arthropod vectors from evolutionary signatures in RNA virus genomes. Science 362, 577–580 (2018). 30385576 10.1126/science.aap9072 PMC6536379

[24] P. W. Ewald, Host-parasite relations, vectors, and the evolution of disease severity. Annu. Rev. Ecol. Syst. 14, 465–485 (1983).

[25] R. M. Anderson, R. M. May, Coevolution of hosts and parasites. Parasitology 85, 411–426 (1982). 6755367 10.1017/s0031182000055360

[26] T. Day, On the evolution of virulence and the relationship between various measures of mortality. Proc. Biol. Sci. 269, 1317–1323 (2002). 12079653 10.1098/rspb.2002.2021 PMC1691045

[27] M. Solignat, B. Gay, S. Higgs, L. Briant, C. Devaux, Replication cycle of chikungunya: A re-emerging arbovirus. Virology 393, 183–197 (2009). 19732931 10.1016/j.virol.2009.07.024 PMC2915564

[28] J. Coffin, R. Swanstrom, HIV pathogenesis: Dynamics and genetics of viral populations and infected cells. Cold Spring Harb. Perspect. Med. 3, a012526 (2013). 23284080 10.1101/cshperspect.a012526 PMC3530041

[29] J. Antonovics , The origin of specificity by means of natural selection: Evolved and nonhost resistance in host-pathogen interactions. Evolution 67, 1–9 (2013). 23289557 10.1111/j.1558-5646.2012.01793.x

[30] P. van Baarlen, A. van Belkum, R. C. Summerbell, P. W. Crous, B. P. H. J. Thomma, Molecular mechanisms of pathogenicity: How do pathogenic microorganisms develop cross-kingdom host jumps? FEMS Microbiol. Rev. 31, 239–277 (2007). 17326816 10.1111/j.1574-6976.2007.00065.x

[31] C. E. Brook, A. P. Dobson, Bats as ‘special’ reservoirs for emerging zoonotic pathogens. Trends Microbiol. 23, 172–180 (2015). 25572882 10.1016/j.tim.2014.12.004 PMC7126622

[32] A. Cogswell-Hawkinson , Tacaribe virus causes fatal infection of an ostensible reservoir host, the Jamaican fruit bat. J. Virol. 86, 5791–5799 (2012). 22379103 10.1128/JVI.00201-12 PMC3347293

[33] G. Kemenesi , Re-emergence of Lloviu virus in *Miniopterus schreibersii* bats, Hungary, 2016. Emerg. Microbes Infect. 7, 66 (2018). 29670087 10.1038/s41426-018-0067-4 PMC5906664

[34] C. Kohl , Zwiesel bat banyangvirus, a potentially zoonotic Huaiyangshan banyangvirus (Formerly known as SFTS)-like banyangvirus in Northern bats from Germany. Sci. Rep. 10, 1370 (2020). 31992832 10.1038/s41598-020-58466-w PMC6987236

[35] M. Staley, C. Bonneaud, Immune responses of wild birds to emerging infectious diseases. Parasite Immunol. 37, 242–254 (2015). 25847450 10.1111/pim.12191

[36] S. P. Thomas, R. A. Suthers, The physiology and energetics of bat flight. J. Exp. Biol. 57, 317–335 (1972).

[37] G. Zhang , Comparative analysis of bat genomes provides insight into the evolution of flight and immunity. Science 339, 456–460 (2013). 23258410 10.1126/science.1230835 PMC8782153

[38] M. Ahn , Dampened NLRP3-mediated inflammation in bats and implications for a special viral reservoir host. Nat. Microbiol. 4, 789–799 (2019). 30804542 10.1038/s41564-019-0371-3 PMC7096966

[39] E. D. Laing , Enhanced autophagy contributes to reduced viral infection in black flying fox cells. Viruses 11, 260 (2019). 10.3390/v11030260 PMC6466025 30875748

[40] J. Xie , Dampened STING-dependent interferon activation in bats. Cell Host Microbe 23, 297–301.e4 (2018). 29478775 10.1016/j.chom.2018.01.006 PMC7104992

[41] G. Goh , Complementary regulation of caspase-1 and IL-1β reveals additional mechanisms of dampened inflammation in bats. Proc. Natl. Acad. Sci. U.S.A. 117, 28939–28949 (2020). 33106404 10.1073/pnas.2003352117 PMC7682399

[42] G. M. Castiglione, Z. Xu, L. Zhou, E. J. Duh, Adaptation of the master antioxidant response connects metabolism, lifespan and feather development pathways in birds. Nat. Commun. 11, 2476 (2020). 32424161 10.1038/s41467-020-16129-4 PMC7234996

[43] W. S. Lai , Life without TTP: Apparent absence of an important anti-inflammatory protein in birds. Am. J. Physiol. Regul. Integr. Comp. Physiol. 305, R689–R700 (2013). 23904106 10.1152/ajpregu.00310.2013 PMC3798794

[44] D. J. Becker, G. Á. Czirják, A. Rynda-Apple, R. K. Plowright, Handling stress and sample storage are associated with weaker complement-mediated bactericidal ability in birds but not bats. Physiol. Biochem. Zool. 92, 37–48 (2019). 30481115 10.1086/701069 PMC7241871

[45] J. L. Geoghegan, E. C. Holmes, The phylogenomics of evolving virus virulence. Nat. Rev. Genet. 19, 756–769 (2018). 30305704 10.1038/s41576-018-0055-5 PMC7096893

[46] S. J. Anthony ; PREDICT Consortium, Global patterns in coronavirus diversity. Virus Evol. 3, vex012 (2017). 28630747 10.1093/ve/vex012 PMC5467638

[47] K. Hampson ; Global Alliance for Rabies Control Partners for Rabies Prevention, Estimating the global burden of endemic canine rabies. PLoS Negl. Trop. Dis. 9, e0003709 (2015). 25881058 10.1371/journal.pntd.0003709 PMC4400070

[48] H. Tian, N. C. Stenseth, The ecological dynamics of hantavirus diseases: From environmental variability to disease prevention largely based on data from China. PLoS Negl. Trop. Dis. 13, e0006901 (2019). 30789905 10.1371/journal.pntd.0006901 PMC6383869

[49] G. Le Flohic, V. Porphyre, P. Barbazan, J.-P. Gonzalez, Review of climate, landscape, and viral genetics as drivers of the Japanese encephalitis virus ecology. PLoS Negl. Trop. Dis. 7, e2208 (2013). 24069463 10.1371/journal.pntd.0002208 PMC3772072

[50] J. L. Geoghegan, E. C. Holmes, Predicting virus emergence amid evolutionary noise. Open Biol. 7, 170189 (2017). 29070612 10.1098/rsob.170189 PMC5666085

[51] R. A. Urbanowicz , Human adaptation of Ebola virus during the West African outbreak. Cell 167, 1079–1087.e5 (2016). 27814505 10.1016/j.cell.2016.10.013 PMC5101188

[52] D. Malvy, A. K. McElroy, H. de Clerck, S. Günther, J. van Griensven, Ebola virus disease. Lancet 393, 936–948 (2019). 30777297 10.1016/S0140-6736(18)33132-5

[53] M. Wille, J. L. Geoghegan, E. C. Holmes, How accurately can we assess zoonotic risk? PLoS Biol. 19, e3001135 (2021). 33878111 10.1371/journal.pbio.3001135 PMC8057571

[54] K. Gruber, Predicting zoonoses. Nat. Ecol. Evol. 1, 98 (2017). 28812659 10.1038/s41559-017-0098 PMC7097299

[55] S. S. Morse , Prediction and prevention of the next pandemic zoonosis. Lancet 380, 1956–1965 (2012). 23200504 10.1016/S0140-6736(12)61684-5 PMC3712877

[56] S. C. Edberg, Global Infectious Diseases and Epidemiology Network (GIDEON): A world wide Web-based program for diagnosis and informatics in infectious diseases. Clin. Infect. Dis. 40, 123–126 (2005). 15614701 10.1086/426549

[57] B. Longdon , The causes and consequences of changes in virulence following pathogen host shifts. PLoS Pathog. 11, e1004728 (2015). 25774803 10.1371/journal.ppat.1004728 PMC4361674

[58] N. Mollentze, D. G. Streicker, P. R. Murcia, K. Hampson, R. Biek, Virulence mismatches in index hosts shape the outcomes of cross-species transmission. Proc. Natl. Acad. Sci. U.S.A. 117, 28859–28866 (2020). 33122433 10.1073/pnas.2006778117 PMC7682402

[59] R Core Team, R: A Language and Environment for Statistical Computing (R Foundation for Statistical Computing, 2018).

[60] J. H. Epstein, H. E. Field, S. Luby, J. R. C. Pulliam, P. Daszak, Nipah virus: Impact, origins, and causes of emergence. Curr. Infect. Dis. Rep. 8, 59–65 (2006). 16448602 10.1007/s11908-006-0036-2 PMC7088631

[61] S. P. Luby , Foodborne transmission of Nipah virus, Bangladesh. Emerg. Infect. Dis. 12, 1888–1894 (2006). 17326940 10.3201/eid1212.060732 PMC3291367

[62] A. Zuur, E. N. Ieno, N. Walker, A. A. Saveliev, G. M. Smith, Mixed Effects Models and Extensions in Ecology with R (Springer-Verlag, 2009).

[63] S. N. Wood, F. Sheipl, Generalized Additive Mixed Models Using “mgcv” and “lme4.” (CRAN, 2020).

[64] G. Marra, S. N. Wood, Practical variable selection for generalized additive models. Comput. Stat. Data Anal. 55, 2372–2387 (2011).

[65] S. Ferrari, F. Cribari-Neto, Beta regression for modelling rates and proportions. J. Appl. Stat. 31, 799–815 (2004).

[66] M. Smithson, J. Verkuilen, A better lemon squeezer? Maximum-likelihood regression with beta-distributed dependent variables. Psychol. Methods 11, 54–71 (2006). 16594767 10.1037/1082-989X.11.1.54

[67] J. I. Marden, Positions and QQ plots. Stat. Sci. 19, 606–614 (2004).

