# Supplementary material for: Bats host the most virulent—but not the most dangerous—zoonotic viruses
Source: Proc Natl Acad Sci U S A. 2022 Mar 29;119(14):e2113628119. doi: 10.1073/pnas.2113628119 (PMC9168486; doi:10.1073/pnas.2113628119)
Supplement: Supplementary File [file pnas.2113628119.sapp.pdf]

Supplementary Material

SI_Data_and_Results. Databases with variable descriptions and references, and table outputs for all selected models

SI_Appendix. Supplementary methods and figures (Figure S1-13)

SI Methods

Exclusion criteria. We excluded seven viruses (Table S2 in *SI Data and Results*) that have only caused human infections in laboratory settings. We additionally did not include viruses such as HIV (1) and HCoV-229E (2) that have zoonotic origins, but have maintained separate, genetically distinct human transmission cycles since before 1950 (Table S3 in *SI Data and Results*). We excluded such viruses for several reasons: precise death and case count records are sparse pre-1950; viruses that have circulated within the human population for centuries or decades often have unconfirmed or disputed origins; and over long timescales, viral evolution in the human population is expected to muddle any relationship between zoonotic history and dynamics in the human population (3). With this strict inclusion criteria, we compiled 89 unique virus species (Table S1 in *SI Data and Results*). Each virus species was associated with one reservoir host order, with the exception of Rabies virus and Mammalian 1 orthobornavirus, which are both known to be maintained by two distinct nonhuman animal reservoir orders in independent transmission cycles (4).

Supplementary analyses. For each virus, in addition to collecting global CFR estimates from the literature, we calculated up to three country-specific CFRs from death and case counts in countries that have reported the largest outbreaks of that virus—when available, using data that spanned multiple outbreaks and/or years to maximize sample size and accuracy. We expected that global CFR estimates would be more precise approximations of virulence, while country-specific CFR reports would allow us to assess and account for potentially confounding effects of regional differences in health care and overall infrastructure. To test whether GDP per-capita predicts country-level variation in CFR, we modeled all 119 country-specific CFR estimates separately (Table S6c in *SI Data and Results*). To gage whether variation in GDP per-capita among viruses' geographic ranges might confound the trends in global CFR estimates, we then modeled GDP per-capita and CFR estimates aggregated at the level of the 86 unique zoonotic transmission chains (Table S6d in *SI Data and Results*). For this second model, we calculated a composite GDP per-capita for each aggregated CFR statistic by weighting each country's GDP per-capita by the proportion of cases in the CFR calculation that were recorded in each country and summing the weighted GDPs per-capita.

We assigned a human transmissibility level of "1" to viruses for which forward transmission in human populations post-spillover had not been recorded; "2" to viruses for which forward transmission in humans had been recorded but was described as atypical; "3" to viruses for which transmission within human populations had occurred regularly but was restricted to self-limiting outbreaks; and "4" to viruses for which endemic human transmission had been reported.

Recording death and case data from laboratory-confirmed outbreaks in the literature required maintaining a strict definition of zoonotic, excluding some viruses that have been included in previous analyses (4–6). We compiled excluded viruses that met looser inclusion

criteria—specifically, seven viruses that have only caused human infections in laboratory settings and 25 viruses that lacked molecular confirmation of infection of humans, but still had serological evidence of infection in humans—in a supplementary database (Table S2 in *SI Data and Results*). To assess whether our observed trends held across a larger sample of zoonotic viruses, we ran an additional GAM analysis of global CFR estimates with this supplementary database, including 121 unique virus species with a total of 126 unique zoonotic transmission chains (Table S6b in *SI Data and Results*). Viruses included in previous analyses that met neither our loose nor strict inclusion criteria are outlined in Table S3 in *SI Data and Results*.

We calculated reservoir host group cophenetic distance from Primates using a composite time-scaled phylogeny of the mean divergence dates for all reservoir clades, as presented in the TimeTree database (7, 61). In our prior analysis (10), phylogenetic distance values were derived from a phylogenetic tree of mammalian cytochrome *b* sequences (3, 62, 63), which captured significantly more variation between host orders. The time-scaled phylogeny used in this analysis produced only six unique distance values across all reservoir groups in our database but represented the only available phylogeny that included both mammals and birds.

Given that the number of zoonoses harbored by a reservoir group appears to correlate with species diversity within that group (7), we hypothesized that species diversity might influence reservoir effect size on CFR in humans; thus, we included reservoir species richness, which we derived from the Catalogue of Life using version 0.9.6 of the taxize library in R (7, 64), taking the sum of values across bird orders for the Aves reservoir group. If increasing a reservoir group's total number of zoonotic viruses also increases their number of virulent zoonoses, reservoir species richness might inflate the mean CFR of zoonotic viruses harbored by species rich reservoir groups—or alternatively, given that most zoonotic viruses have low CFRs in humans, species richness might instead reduce the mean CFR associated with these reservoirs. Nevertheless, we expected that higher numbers of zoonotic virus species would inflate the total death burdens associated with species rich reservoir groups.

SI Figures

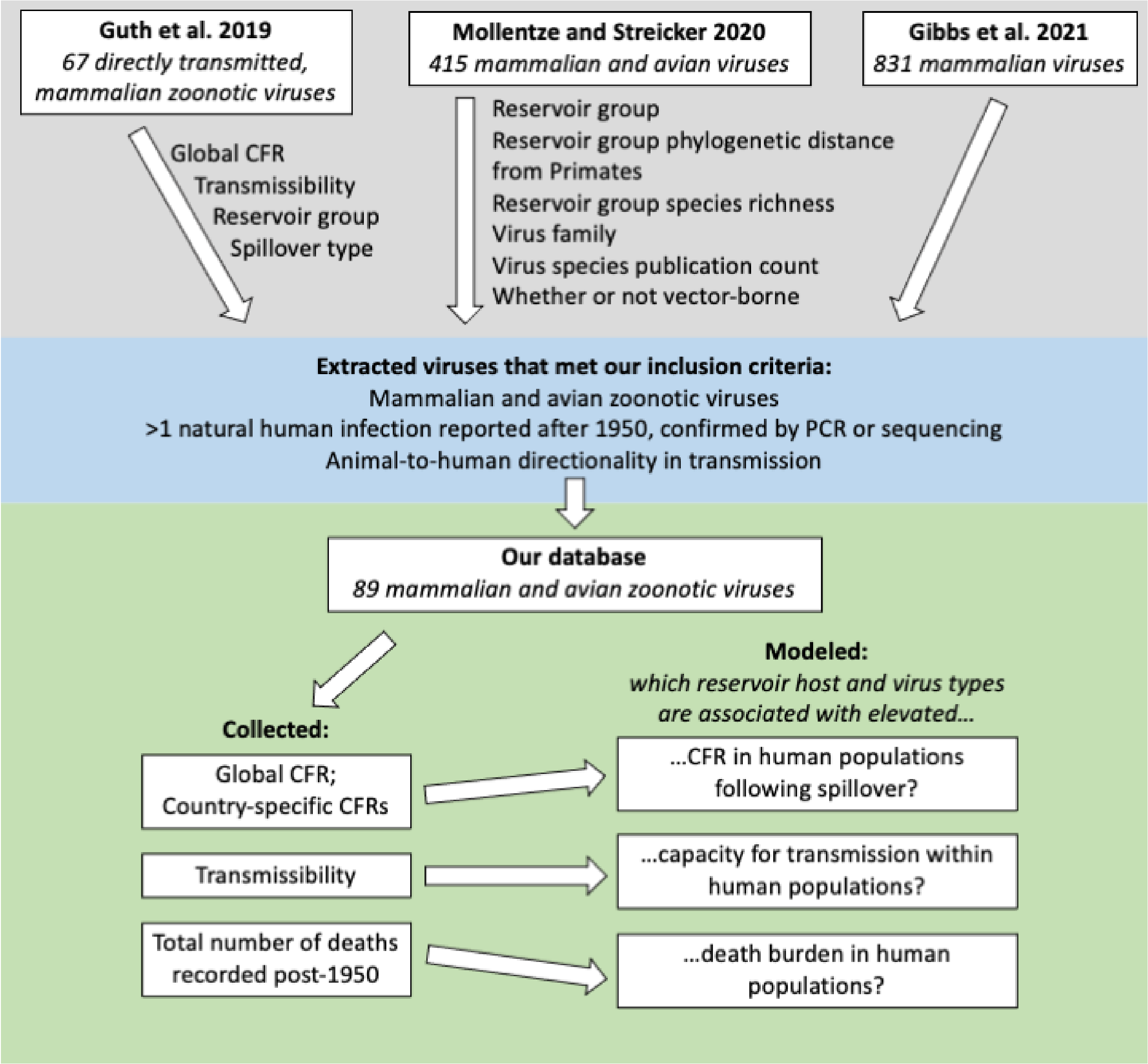

Figure S1. Data provenance and analysis flowchart.

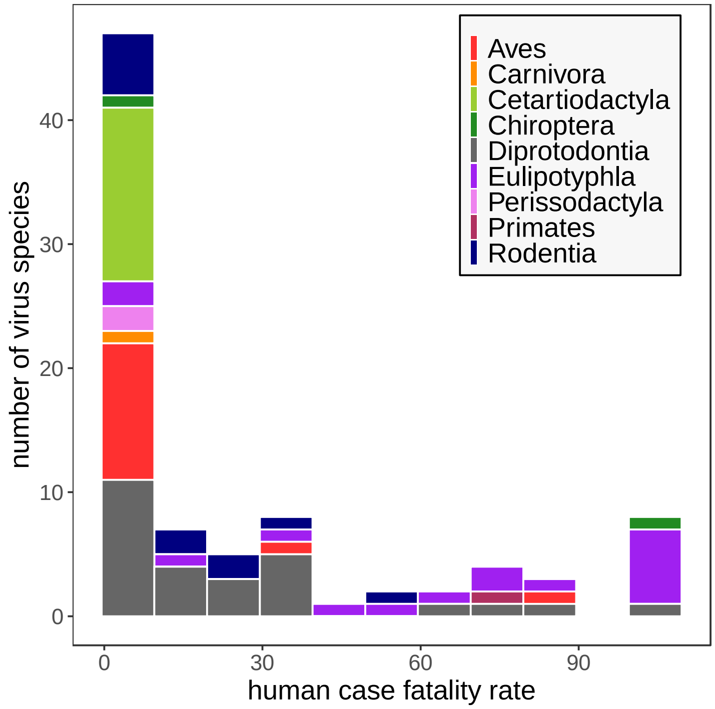

Figure S2. Histogram of human case fatality rates (CFRs) across all zoonotic virus species included in our virulence analysis, grouped by reservoir host type.

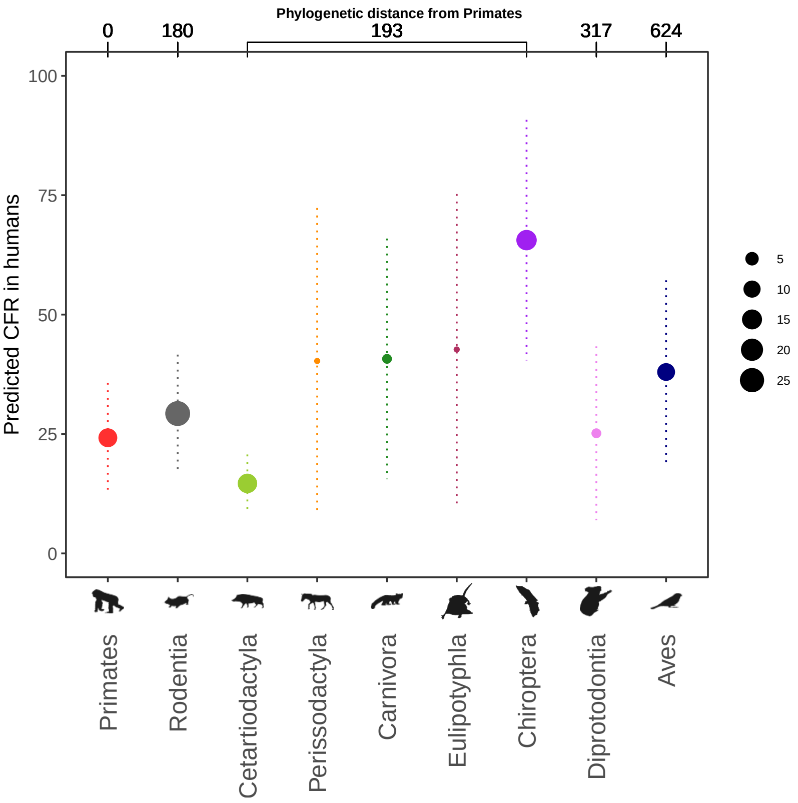

Figure S3. Predicted human CFR of zoonotic viruses sourced from each reservoir host group when using the top selected model of global CFR estimates.

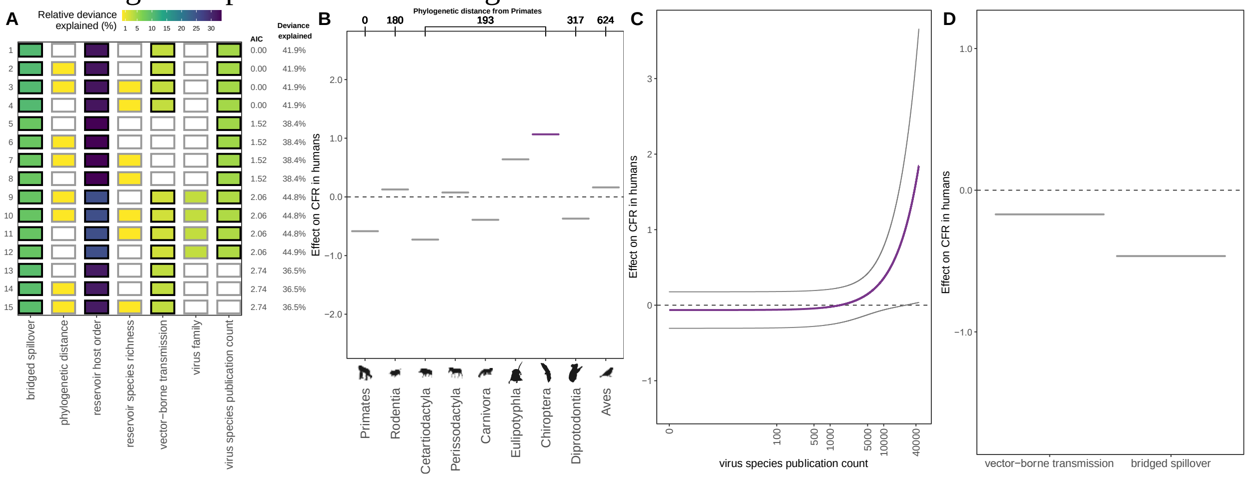

Figure S4. Predictors of global CFR estimates, excluding bat lyssaviruses. (A) Top 15 models ranked by AIC. Rows represent individual models and columns represent predictor variables. Cells are shaded according to the proportion of deviance explained by each predictor. Cells representing predictor variables with a p-value significance level of <0.1 are outlined in black and otherwise outlined in gray. (B-D) Effects present in the top model: reservoir host group, log-transformed virus species publication count, vector-borne transmission, and bridged spillover. Lines represent the predicted effect of the x-axis variable when all other variables are held at their median value (if numeric) or their mode (if categorical). Shaded regions indicate 95% CIs by standard error and points represent partial residuals. An effect is shaded in gray if the 95% CI crosses zero across the entire range of the predictor variable; in contrast, an effect is shaded in

purple and considered “significant” if the 95% CI does not cross zero. Full model results are outlined in Table S6a in *SI Data and Results*. (B) Reservoir host groups are ordered by increasing cophenetic phylogenetic distance from Primates (in millions of years), as indicated on the top axis.

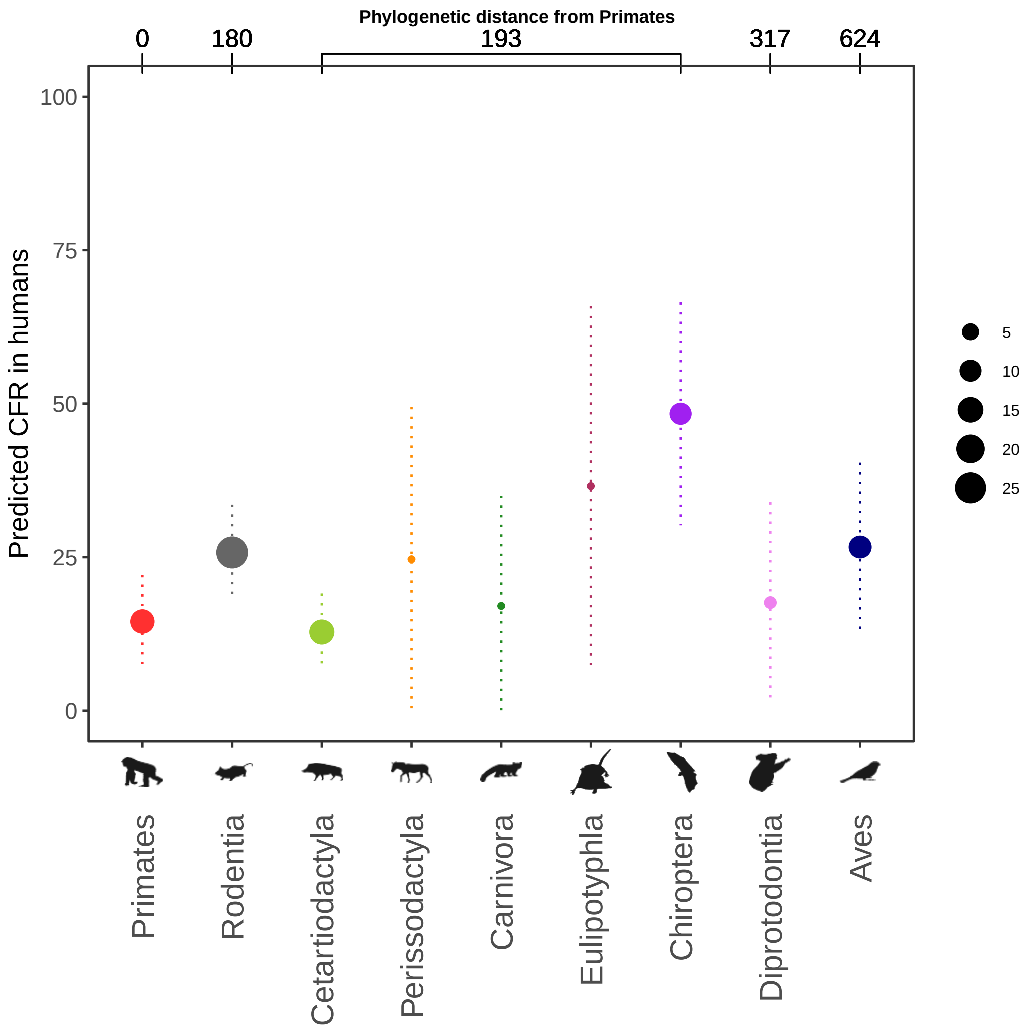

Figure S5. Predicted human CFR of zoonotic viruses sourced from each reservoir host group when using the top selected model of global CFR estimates, excluding bat lyssaviruses.

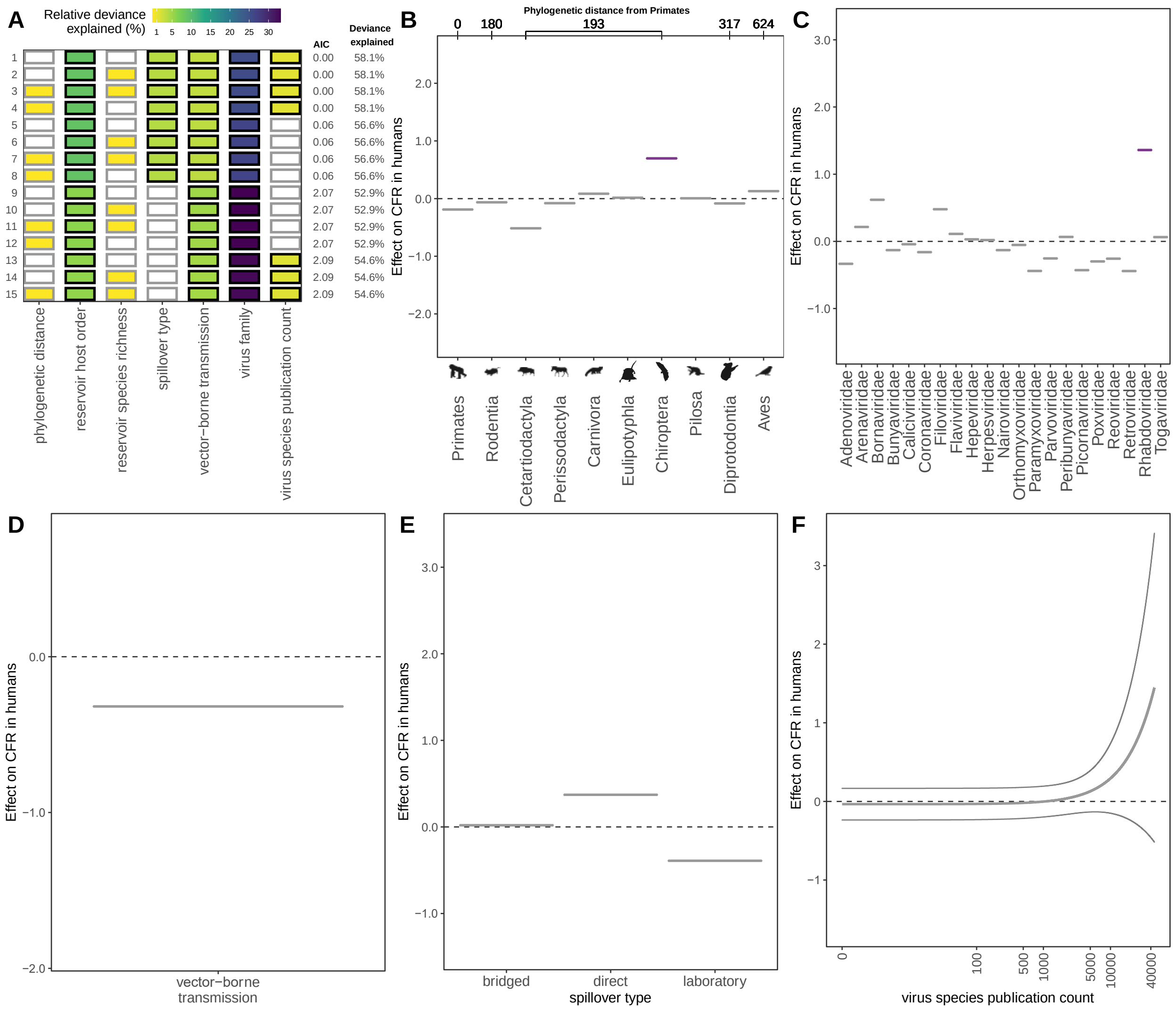

Figure S6. Predictors of global CFR estimates, including virus species that met a lenient definition of zoonotic. (A) Top 15 models ranked by AIC. Rows represent individual models and columns represent predictor variables. Cells are shaded according to the proportion of deviance explained by each predictor. Cells representing predictor variables with a p-value significance level of <0.1 are outlined in black and otherwise outlined in gray. (B-F) Effects present in the top model: reservoir host group, virus family, vector-borne transmission, spillover type, and virus species publication count. Lines represent the predicted effect of the x-axis variable when all other variables are held at their median value (if numeric) or their mode (if categorical). Shaded regions indicate 95% CIs by standard error and points represent partial residuals. An effect is shaded in gray if the 95% CI crosses zero across the entire range of the predictor variable; in contrast, an effect is shaded in purple and considered "significant" if the 95% CI does not cross zero. Full model results are outlined in Table S6b in *SI Data and Results*. (B) Reservoir host groups are ordered by increasing phylogenetic distance from Primates, as indicated on the top axis.

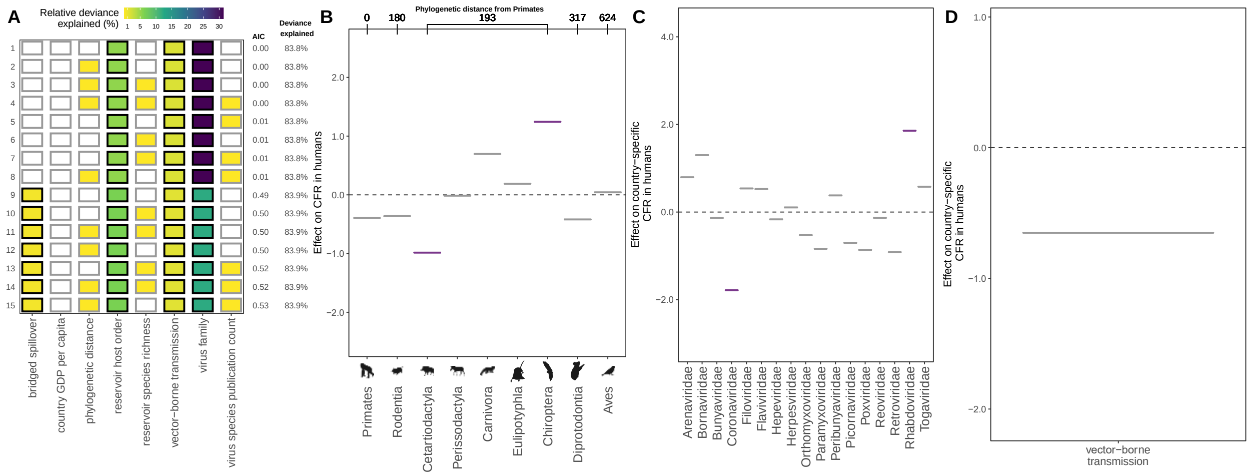

Figure S7. Predictors of variation among the 119 country-specific CFR estimates. (A) Top 15 models ranked by AIC. Rows represent individual models and columns represent predictor variables. Cells are shaded according to the proportion of deviance explained by each predictor. Cells representing predictor variables with a p-value significance level of <0.1 are outlined in black and otherwise outlined in gray. (B-D) Effects present in the top model: reservoir host group, virus family, and vector-borne transmission. Lines represent the predicted effect of the x-axis variable when all other variables are held at their median value (if numeric) or their mode (if categorical). Shaded regions indicate 95% CIs by standard error and points represent partial residuals. An effect is shaded in gray if the 95% CI crosses zero across the entire range of the predictor variable; in contrast, an effect is shaded in purple and considered "significant" if the 95% CI does not cross zero. Full model results are outlined in Table S6c in *SI Data and Results*. (B) Reservoir host groups are ordered by increasing cophenetic phylogenetic distance from Primates (in millions of years), as indicated on the top axis.

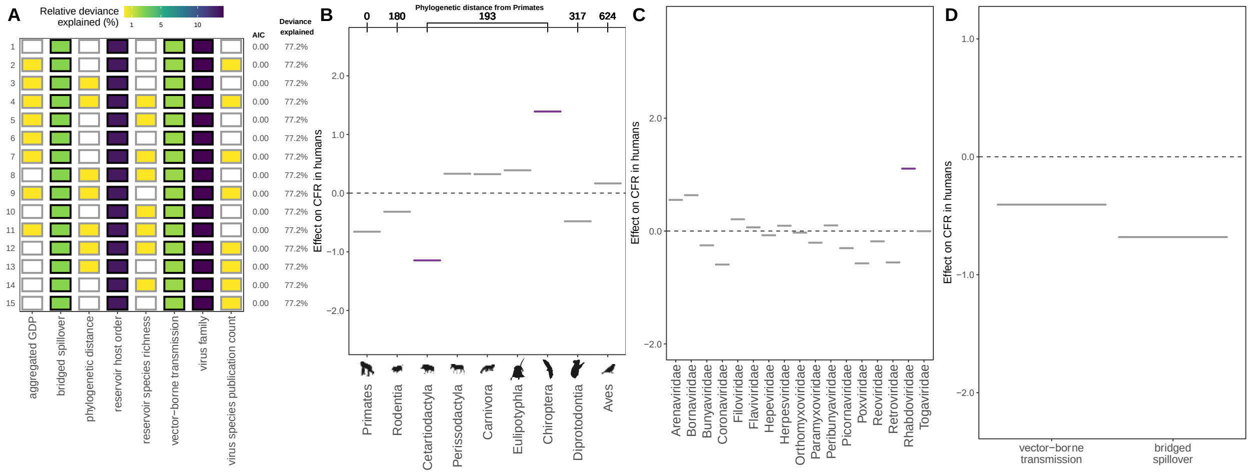

Figure S8. Predictors of CFRs calculated from country-level data aggregated at the level of the 86 unique zoonotic transmission chains. (A) Top 15 models ranked by AIC. Rows represent individual models and columns represent predictor variables. Cells are shaded according to the proportion of deviance explained by each predictor. Cells representing predictor variables with a p-value significance level of <0.1 are outlined in black and otherwise outlined in gray. (B-D) Effects present in the top model: reservoir host group, virus family, vector-borne transmission, and bridged spillover. Lines represent the predicted effect of the x-axis variable when all other

variables are held at their median value (if numeric) or their mode (if categorical). Shaded regions indicate 95% CIs by standard error and points represent partial residuals. An effect is shaded in gray if the 95% CI crosses zero across the entire range of the predictor variable; in contrast, an effect is shaded in purple and considered "significant" if the 95% CI does not cross zero. Full model results are outlined in Table S6d in *SI Data and Results*. (B) Reservoir host groups are ordered by increasing cophenetic phylogenetic distance from Primates (in millions of years), as indicated on the top axis.

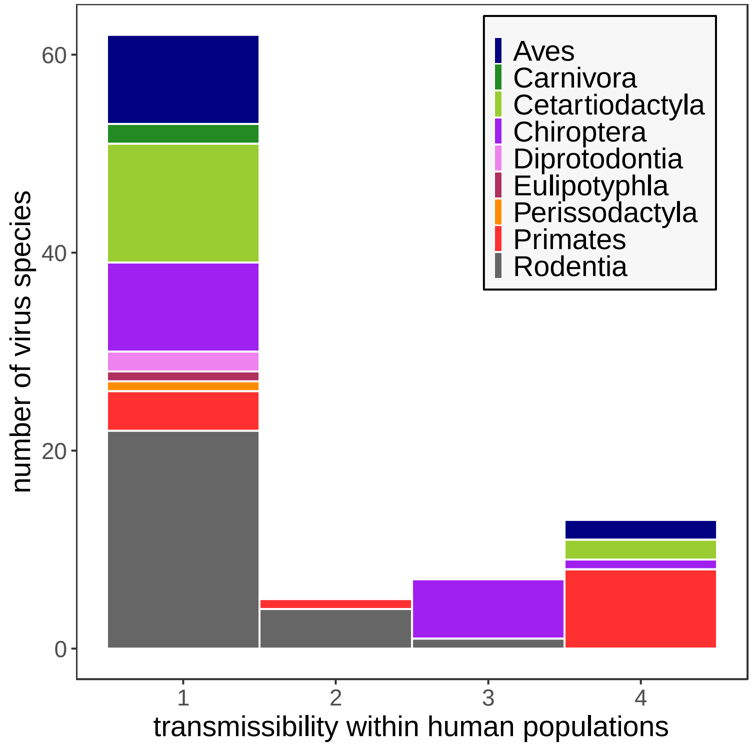

Figure S9. Histogram of human transmissibility rankings across all zoonotic virus species included in our analysis of capacity for forward transmission in humans, grouped by host order.

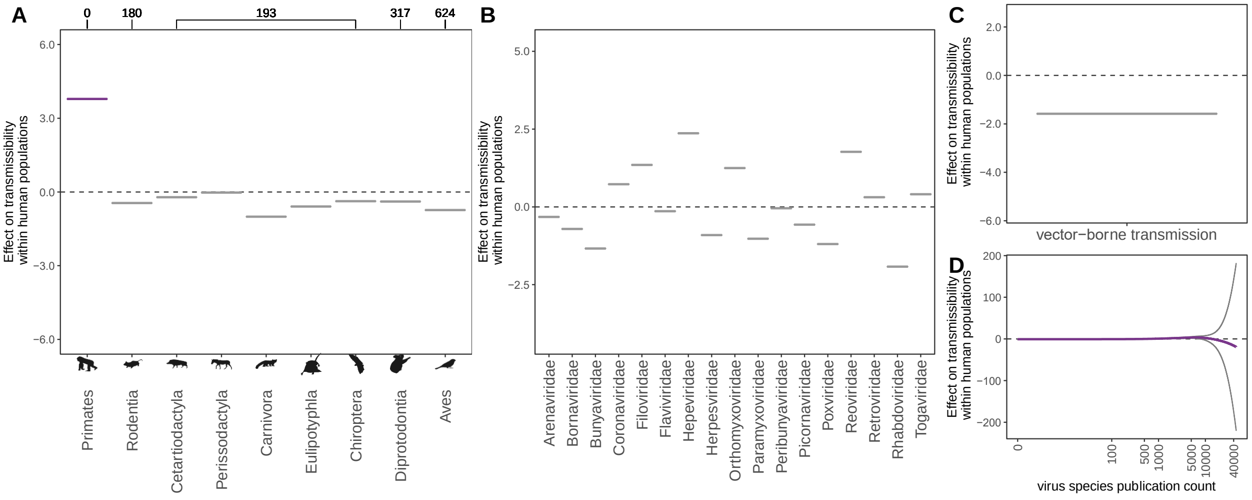

Figure S10. Effects present in the selected model to predict capacity for forward transmission within the human population with reservoir host order as a predictor instead of phylogenetic

distance from humans. Effects include reservoir host group, virus family, vector-borne transmission, and virus species publication count. Lines represent the predicted effect of the x-axis variable when all other variables are held at their median value (if numeric) or their mode (if categorical). Shaded regions indicate 95% CIs by standard error and points represent partial residuals. An effect is shaded in gray if the 95% CI crosses zero across the entire range of the predictor variable; in contrast, an effect is shaded in purple and considered "significant" if the 95% CI does not cross zero. Full model results are outlined in Table S6e in *SI Data and Results*. (A) Reservoir host groups are ordered by increasing cophenetic phylogenetic distance from Primates (in millions of years), as indicated on the top axis.

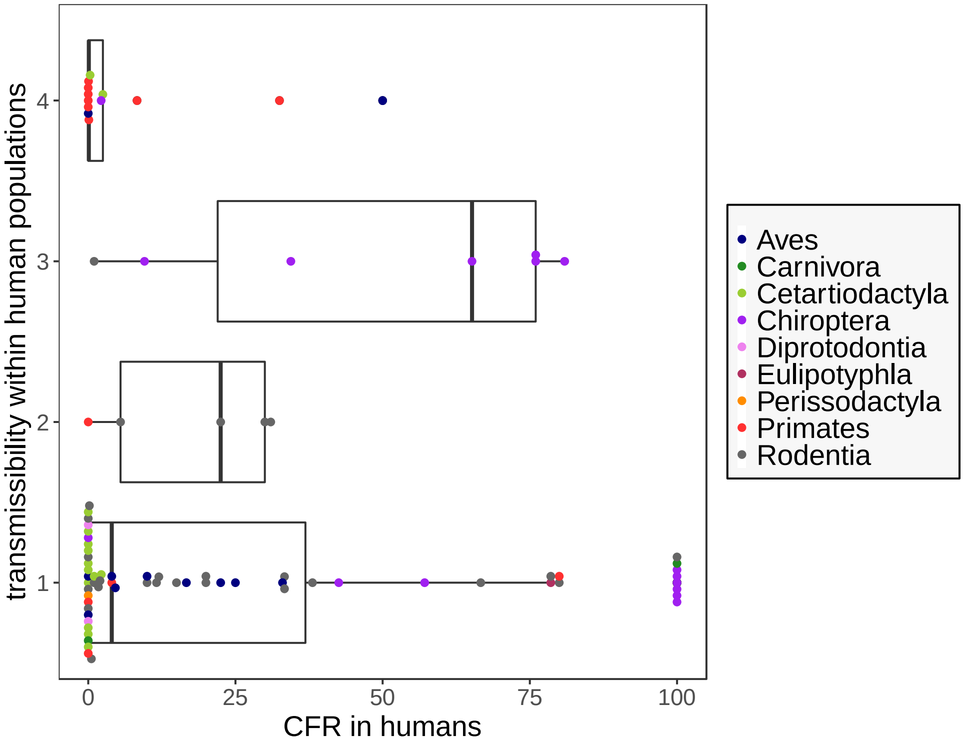

Figure S11. Relationship between CFR and transmissibility in humans.

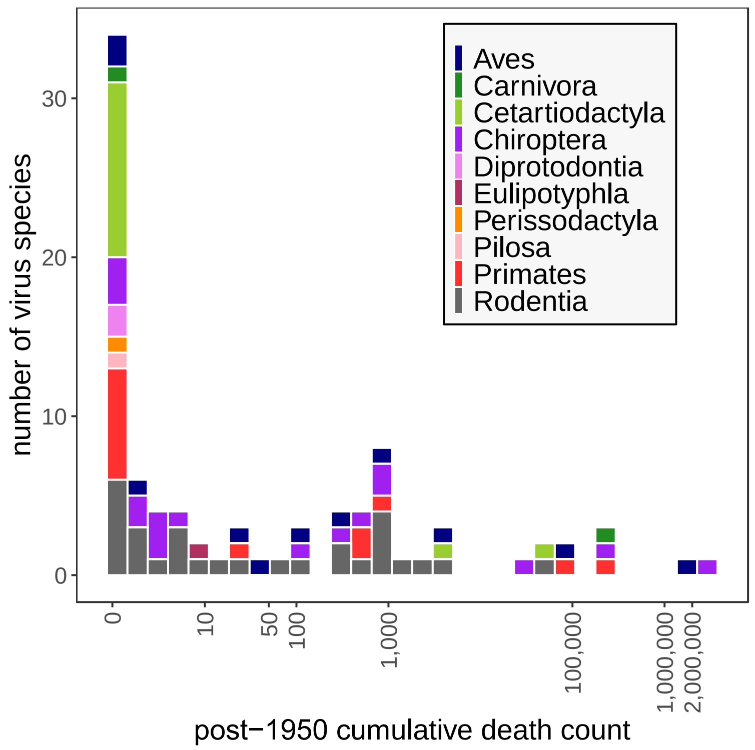

Figure S12. Histogram of post-1950 death counts, grouped by host order. Here, death counts were plotted to display the data distribution and are not adjusted for variation in the length of the reporting timeline. In the death burden model, we normalized counts by including an offset for the exact number of years over which deaths were recorded.

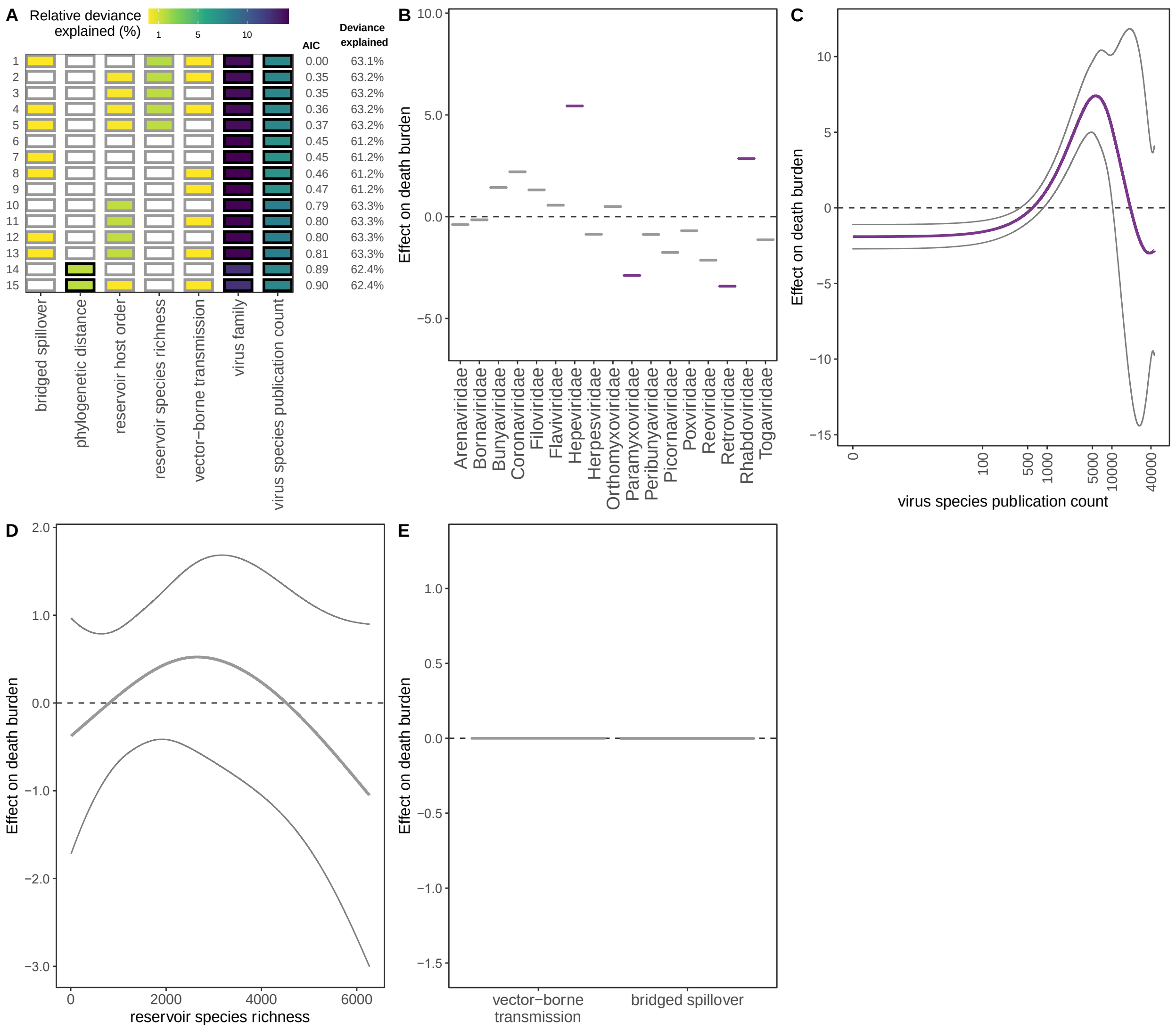

Figure S13. Predictors of post-1950 death burden. (A) Top 15 models ranked by AIC. Rows represent individual models and columns represent predictor variables. Cells are shaded according to the proportion of deviance explained by each predictor. Cells representing predictor variables with a p-value significance level of <0.1 are outlined in black and otherwise outlined in gray. (B-E) Effects present in the top model: virus family, virus species publication count, reservoir group species richness, vector-borne transmission, and bridged spillover. Lines represent the predicted effect of the x-axis variable when all other variables are held at their median value (if numeric) or their mode (if categorical). Shaded regions indicate 95% CIs by standard error and points represent partial residuals. An effect is shaded in gray if the 95% CI crosses zero across the entire range of the predictor variable; in contrast, an effect is shaded in purple and considered "significant" if the 95% CI does not cross zero. Full model results are outlined in Table S6f in *SI Data and Results*. (C) Reservoir host groups are ordered by increasing cophenetic phylogenetic distance from Primates (in millions of years), as indicated on the top axis.

References

1. J. Hemelaar, The origin and diversity of the HIV-1 pandemic. *Trends Mol. Med.* **18**, 182–192 (2012).

2. Z.-W. Ye, *et al.*, Zoonotic origins of human coronaviruses. *Int. J. Biol. Sci.* **16**, 1686–1697 (2020).

3. J. L. Geoghegan, E. C. Holmes, Predicting virus emergence amid evolutionary noise. *Open Biol.* **7**, 170189 (2017).

4. N. Mollentze, D. G. Streicker, Viral zoonotic risk is homogenous among taxonomic orders of mammalian and avian reservoir hosts. *Proc. Natl. Acad. Sci.* **117**, 9423–9430 (2020).

5. K. J. Olival, *et al.*, Host and viral traits predict zoonotic spillover from mammals. *Nature* **546**, 646–650 (2017).

6. L. Brierley, A. B. Pedersen, M. E. J. Woolhouse, Tissue tropism and transmission ecology predict virulence of human RNA viruses. *PLOS Biol.* **17**, e3000206 (2019).
